# Single‐Cell Transcriptomics Reveals ITGA2‐Mediated Metabolic Reprogramming and Immune Crosstalk in Pediatric Thyroid Carcinogenesis

**DOI:** 10.1002/advs.202504088

**Published:** 2025-09-23

**Authors:** Zhi‐jun Zhan, Ning Li, Yan Sun, Lu Chen, Jun‐da Yin, Yun‐han Shan, Jia‐xing Zeng, Zhe‐cheng Li, Hai‐long Tan, Neng Tang, Shi Chang, Peng Huang

**Affiliations:** ^1^ Department of General Surgery XiangYa Hospital Central South University Changsha Hunan 410008 China; ^2^ Clinical Research Center for Thyroid Disease in Hunan Province Xiangya Hospital Central South University Changsha Hunan 410008 China; ^3^ Hunan Provincial Engineering Research Center for Thyroid and Related Diseases Treatment Technology Xiangya Hospital Central South University Changsha Hunan 410008 China; ^4^ National Clinical Research Center for Geriatric Disorders Xiangya Hospital Central South University Changsha Hunan 410008 China

**Keywords:** glycolysis, heterogeneity, landscape, macrophages, pediatric thyroid carcinoma

## Abstract

Pediatric papillary thyroid carcinoma (PPTC) has exhibited a progressive increase in incidence in recent years, characterized by heightened biological aggressiveness relative to adult papillary thyroid carcinoma (APTC). Nevertheless, the molecular mechanisms governing PPTC‐specific pathobiology remain elusive. Through high‐resolution single‐cell RNA sequencing analysis of 90234 cellular transcriptomes from 4 PPTC and 9 APTC clinical specimens, a phenotypically distinct cellular subpopulation (ITGA2^hi^‐PTC cells) mechanistically responsible for PPTC's distinct clinical behavior, is identified. By integrating PPTC‐derived organoid models with in vivo functional validation and multiplex immunohistochemistry, it is demonstrated that ITGA2 orchestrates dual oncogenic pathways: 1) augmentation of glycolytic flux and 2) induction of M2 macrophage polarization. These synergistic mechanisms fundamentally drive PPTC oncogenesis and metastatic progression. Cross‐validation across independent clinical cohorts consistently confirms the translational significance of these findings. Our multi‐omics characterization of PPTC‐specific cellular ecosystems and signaling cascades establishes a mechanistic framework for advancing diagnostic precision and targeted therapeutic development.

## Graphical abstract

This study unveils ITGA2⁺ tumor cells as key drivers of pediatric thyroid cancer aggressiveness. These cells orchestrate dual oncogenic pathways: GLUT1‐mediated glycolytic reprogramming and M2 macrophage polarization. This metabolic‐immunological crosstalk promotes tumor progression and metastasis. Multi‐platform validation establishes ITGA2 as a pivotal therapeutic target, advancing precision oncology for this childhood malignancy.

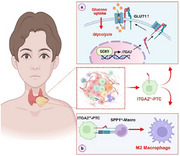

## 1 Introduction

Thyroid carcinoma represents the most prevalent endocrine malignancy in pediatric populations, with incidence rates demonstrating a two‐fold increase over the past four decades.^[^ ^1^, ^2^ ^]^ Substantial epidemiological evidence identifies ionizing radiation exposure, particularly during early childhood (<5 years), as the principal etiological factor for pediatric thyroid carcinogenesis.^[^ ^3^, ^4^ ^]^ In response to these findings, international radiation protection protocols were instituted in 2002 to minimize pediatric exposure to diagnostic and therapeutic radiation.^[^ ^5^ ^]^ Paradoxically, epidemiological surveillance has documented persistent increases in pediatric thyroid cancer incidence during subsequent decades,^[^ ^1^, ^6^ ^]^ underscoring critical knowledge gaps in understanding its pathogenic mechanisms.

While pediatric differentiated thyroid carcinomas exhibit histological similarities to adult counterparts, with papillary thyroid carcinoma (PTC) predominating in both cohorts,^[^ ^7^ ^]^ clinical manifestations diverge substantially. Compared to adult PTC (APTC), pediatric PTC (PPTC) demonstrates more aggressive biological behavior characterized by larger primary tumors, bilateral involvement, extrathyroidal extension (ETE), higher rates of lymphatic and pulmonary metastases, and higher recurrence rates.^[^ ^8^ ^]^ These clinical disparities suggest distinct tumorigenic pathways underlying PPTC, necessitating a comprehensive investigation of the intrinsic mechanisms driving its aggressive phenotype.

Emerging molecular evidence underscores distinct genetic landscapes between pediatric (PPTC) and adult (APTC) papillary thyroid carcinomas. While activating BRAF V600E mutations are highly prevalent in APTC (occurring in up to 60% of cases), they are substantially less common in PPTC (≈25%).^[^ ^9^, ^10^ ^]^ Conversely, driver gene rearrangements and fusions—particularly RET rearrangements (e.g., RET/PTC1, RET/PTC3; ≈30%–50%) and NTRK fusions (e.g., ETV6‐NTRK3; ≈10%–20%)—are significantly enriched in the pediatric cohort.^[^ ^9^, ^11^ ^]^ Furthermore, PPTCs rarely harbor coexisting driver mutations in the telomerase reverse transcriptase (TERT) promoter or tumor protein p53 (TP53) genes, alterations strongly associated with aggressive disease behavior and adverse outcomes in APTC.^[^ ^12^ ^]^ However, conventional molecular profiling has proven insufficient to fully explain PPTC's unique clinicopathological characteristics, potentially reflecting complex interactions within the tumor microenvironment (TME), encompassing intratumoral heterogeneity and cancer‐stromal‐immune crosstalk. Recent advancements in single‐cell RNA sequencing (scRNA‐seq) technology have enabled high‐resolution characterization of cellular ecosystems in APTC, revealing previously unrecognized tumor subpopulations and complex TME dynamics.^[^ ^13^, ^14^ ^]^ These methodological breakthroughs position scRNA‐seq as an essential tool for deciphering PPTC biology.

This study employed scRNA‐seq to systematically characterize cellular heterogeneity and TME dynamics in PPTC pathogenesis. We identified a novel tumor subpopulation (ITGA2^hi^‐PTC cells) implicated in driving PPTC's aggressive phenotype. Experimental validation through in vitro models and multiplex immunohistochemical (IHC) analyses, supplemented by clinical cohort verification, confirmed these findings. Our multi‐modal approach elucidates critical molecular pathways and cellular interactions in PPTC, providing a foundation for developing targeted diagnostic and therapeutic strategies.

## 2 Results

### 2.1 Single‐Cell Transcriptomic Atlas and Cell Typing in PPTC and APTC

**Figure 1:**
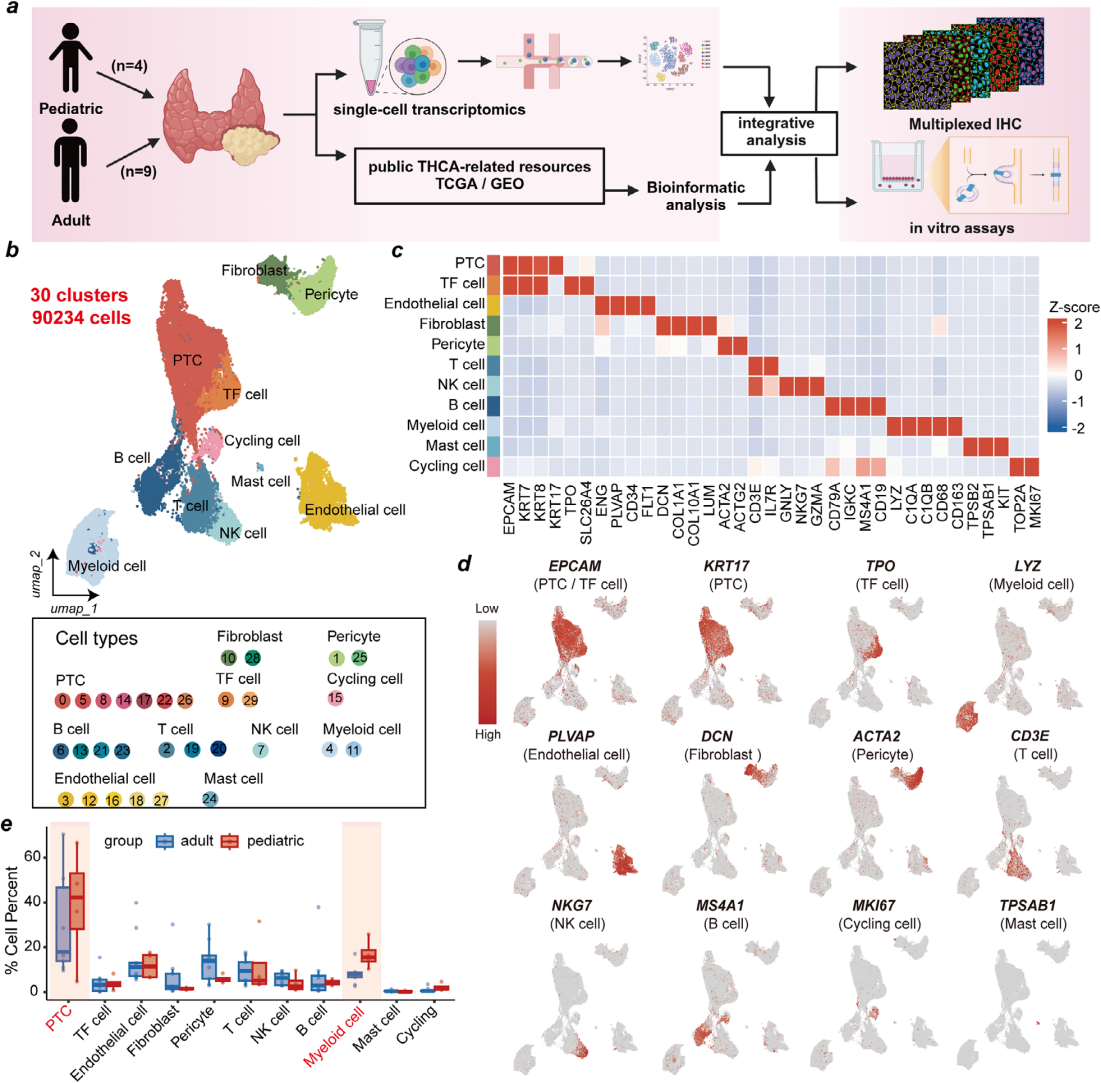
Single‐cell transcriptomic analysis in pediatric and adult PTC tissues. **a)** Workflow of sample collection and data analysis in this study. **b)** Uniform Manifold Approximation and Projection (UMAP) visualization of 90234 cells from 13 samples, showing 30 clusters in different colors. **c)** Heatmap showing genes (columns) that were differentially expressed across various cell types (rows). Scale bar depicting low expression in blue and high expression in orange. **d)** UMAP plots showing the expression profiles of indicated cell‐type‐specific marker genes, color gradient indicates log normalized expression levels (gray means no or low, red means high). **e)** Bar plot showing the cell type abundance for samples from adult (*n* = 9) and pediatric group (*n* = 4), as measured by scRNA‐seq data in this study, data are presented as mean ± SEM. UMAP, Uniform Manifold Approximation and Projection. PTC, Papillary thyroid carcinoma. scRNA‐seq, single‐cell RNA sequencing.

Due to the scarcity of pediatric PTC biospecimens suitable for scRNA‐Seq, we prioritized 4 treatment‐naïve samples from children and 9 samples from adults for the control group. While this limits population‐level inferences, it enables unprecedented dissection of cellular heterogeneity within individual tumors (**Figure** **1**a). The clinicopathological characteristics of these patients are presented in Table S1 and Figure S1 (Supporting Information).

After strict quality control and filtration, a total of 90234 high‐quality single cells, expressing 32906 genes, were retained for subsequent analysis. Uniform manifold approximation and projection (UMAP) revealed thirty distinct cell clusters (Figure 1b; Figure S2a, Supporting Information). Eleven major cell types were annotated based on the expression of canonical marker genes: PTC cells (*n *= 30559) were identified as cells expressing EPCAM, KRT7, KRT8 and KRT17 (clusters 0, 5, 8, 14, 17, 22, 26); thyroid follicular (TF) cells (*n* = 4407) were identified as cells expressing TPO and SLC26A4 (clusters 9, 29); endothelial cells (*n* = 11348) were identified as cells expressing ENG, PLVAP, CD34 and FLT1 (clusters 3, 12, 16, 18, 27); fibroblasts (*n* = 3412) were identified as cells positive for COL1A1, COL10A1, DCN and LUM (clusters 10, 28); pericyte cells (*n* = 8550) were identified as those expressing ACTA2 and ACTG2 (clusters 1, 25); T cells (*n* = 8785) were identified as cells expressing CD3E and IL7R (clusters 2, 19, 20); NK cells (*n* = 4707) were identified as cells expressing GNLY, GZMA, and NKG7 (cluster 7); B cells (*n* = 7211) were identified as cells expressing CD79A, IGKC, MS4A1 and CD19 (clusters 6, 13, 21, 23); myeloid cells (*n* = 9737) were identified as cells expressing CD68, CD163, LYZ, C1QA, and C1QB (cluster 4, 11); mast cells (*n* = 276) were identified as cells expressing TPSB2, TPSAB1 and KIT (cluster 24); and cycling cells (*n* = 1242) were identified as cells expressing MKI67 and TOP2A (cluster 15) (Figure 1c). We confirmed the high specificity and expression levels of these canonical markers for their respective cell types (Figure 1d). To explore differences in TME composition between PPTC and APTC, we compared the relative proportions of each cell type. PPTC samples exhibited significantly higher proportions of PTC cells and myeloid cells compared to APTC samples, whereas the proportions of T cells and NK cells were significantly lower (Figure 1e; Figure S2b,c, Supporting Information). These pronounced differences in cellular composition underscore the distinct TME landscape of PPTC, which was further investigated in our subsequent analyses.

### 2.2 High Heterogeneity of ITGA2^hi^‐PTC Cells Between PPTC and APTC

**Figure 2:**
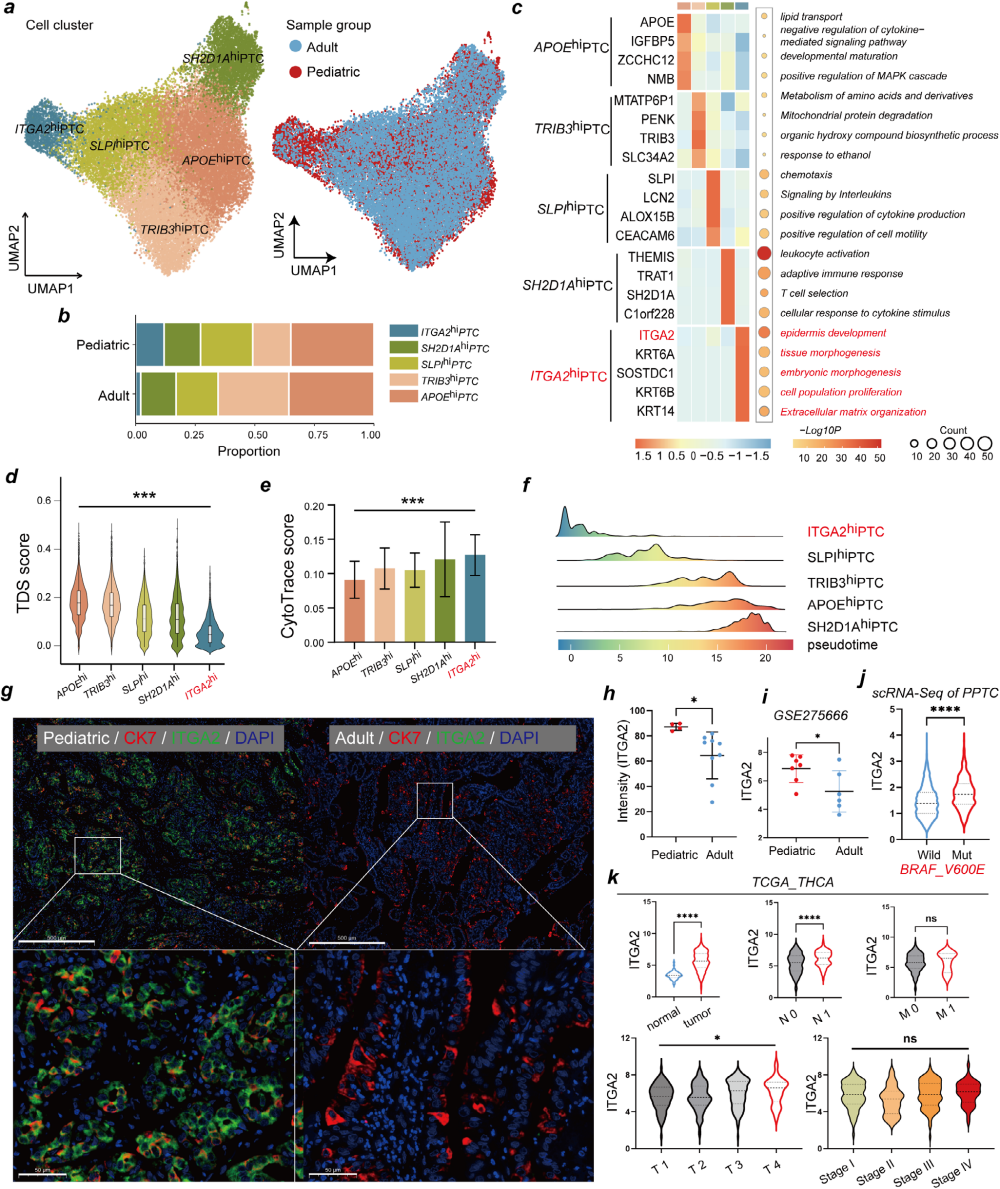
Functional characterization and evolutionary trajectories of distinct PTC subclusters. **a)** UMAP plots showing the distribution of PTC cell subtypes (left) and different sample groups (right). **b)** Bar plot showing the proportion of these annotated subclusters originating from pediatric and adult patients. **c)** Heatmap (left) displaying Z‐score normalized expression of cluster‐specific genes (Scale bar ranging from −1.5 to 1.5), and dot plot showing (right) the corresponding enriched biological functions of these annotated PTC subclusters. Significance was determined using a two‐sided Wilcoxon rank‐sum test. **d)** Box plots illustrating the TDS scores, and **e)** Bar plot illustrating the CytoTrace scores between APOE^hi^‐PTC cells (*n* = 10, 665), TRIB3^hi^‐PTC cells (*n* = 7, 175), SLPI^hi^‐PTC cells (*n* = 5, 863), SH2D1A^hi^‐PTC cells (*n* = 4, 531), and ITGA2^hi^‐PTC cells (*n* = 1921), significance was determined using a Kruskal‐Wallis test, ^***^ *p *< 0.001. **f)** Ridgeline plot showing the dynamic changes in PTC cell subtypes along the pseudotime. **g)** Representative images of multiplex immunohistochemistry (mIHC) staining of ITGA2^hi^‐PTC cells in pediatric and adult PTC samples. Scale bar = 500 µm. **h)** Quantitative analysis of immunofluorescence staining for ITGA2 in pediatric (*n* = 4) and adult group (*n* = 9), Two‐sided student's t test, ^*^ *p *< 0.05. **i)** ITGA2 expression in pediatric PTC (*n* = 7) and adult PTC (*n* = 6) tissues, data from GSE275666, Two‐sided student's t test, ^*^ *p *< 0.05. **j)** Comparison of ITGA2 expression levels between BRAFV600E‐mutant PTC cells (*n* = 3345) and wild‐type PTC cells (*n* = 9662) in scRNA‐seq data of PPTC, Two‐sided student's t test, ^****^ *p *< 0.0001. **k)** Correlations of ITGA2 genes expression and various pathological factors of PTC in the TCGA cohort, (upper left panel) tumor (*n* = 512) versus normal thyroid (*n* = 337), (central upper panel) N0 (*n* = 186) versus N1(*n* = 191), (upper right panel) M0 (*n* = 272) versus M1 (*n* = 8), (lower left panel) T‐stages (n (T1)=110, n(T2)=130, n(T3)=149, n(T4)=20), (lower right panel) pathological stage (n(stage I)=231, n(stage II)=37, n(stage III)=100, n(stage IV)=43). Two‐sided student's t test or one‐way ANOVA test with Tukey's post hoc test, ^*^ *p *< 0.05, ^****^ *p *< 0.0001, ns means no significance. TDS, thyroid differentiation score. THCA, thyroid carcinoma. TCGA, The Cancer Genome Atlas. GEPIA, Gene Expression Profiling Interactive Analysis.

To decipher the landscape of tumor cells, following the removal of 404 doublets, 30155 PTC cells were regrouped into five subclusters, including APOE^hi^‐PTC, TRIB3^hi^‐PTC, SLPI^hi^‐PTC, SH2D1A^hi^‐PTC, and ITGA2^hi^‐PTC cells, defined by a unique subset of highly expressed genes in each subcluster (**Figure** **2**a; Table S2, Supporting Information). These subclusters presented unique gene expression patterns and biological functions, and their proportions varied significantly between PPTC and APTC patients, indicating high intertumor heterogeneity (Figure 2a–c). ITGA2^hi^‐PTC cells were increased in PPTC samples and exhibited high expression of ITGA2, KRT6A, SOSTDC1, KRT6B, and KRT14, genes associated with tumor progression (Figure 2c **;** Table S3, Supporting Information). Furthermore, ITGA2^hi^‐PTC cells exhibited high functional enrichment scores for cell proliferation and extracellular matrix organization (Figure 2c).

To expand the sample size, we acquired scRNA‐seq data from one adult and five pediatric PTC samples through the GEO database (GSE281736). Following quality control filtering, PTC cells underwent clustering analysis, yielding 13 distinct cellular subclusters (Figure S3a,b, Supporting Information). Notably, subcluster 11 demonstrated significant enrichment in pediatric PTC samples, and its gene expression profile closely matched that of the ITGA2^hi^‐PTC cell subcluster identified in this study. Consequently, subcluster 11 was designated as the ITGA2^hi^‐PTC cell subcluster (Figure S3c–f, Supporting Information). These findings suggest that the ITGA2^hi^‐PTC cell subpopulation may play a pivotal role in the pathological progression of pediatric PTC.

To further validate these findings, we characterized the differences among the five cell subpopulations using three complementary approaches. First, we calculated the thyroid differentiation score (TDS), a widely used metric to evaluate the differentiation status of PTC thyrocytes.^[^ ^12^ ^]^ ITGA2^hi^‐PTC cells exhibited the lowest TDS values among the subclusters, which suggested that this group of cells had a lower degree of differentiation than the other groups did (Figure 2d; Table S4, Supporting Information). Second, using the CytoTRACE algorithm to assess differentiation potential, we found that ITGA2^hi^‐PTC cells had elevated CytoTRACE scores, indicating increased cell stemness and proliferation rates (Figure 2e; Table S4, Supporting Information). Third, we employed the Monocle3 algorithm to reconstruct the pseudotime trajectory and infer the dynamic evolution among PTC subclusters. Trajectory analysis positioned ITGA2^hi^‐PTC cells at the origin of the trajectory, with SLPI^hi^‐PTC cells primarily representing intermediate states, and APOE^hi^‐PTC, SH2D1A^hi^‐PTC, and TRIB3^hi^‐PTC cells comprising terminally differentiated states (Figure 2f; Figure S4a, Supporting Information). Collectively, these data indicate that ITGA2^hi^‐PTC cells represent a progenitor‐like population in PPTC that may differentiate into other PTC cell clusters exhibiting functions such as lipid metabolism, amino acid metabolism, and immune regulation (Figure S4b–d, Supporting Information).

#### 2.2.1 ITGA2 is Highly Expressed in PPTC and is Associated With the Invasive and Metastatic Phenotypes

We then utilized multiplex IHC to further validate the differential distribution of ITGA2^hi^‐PTC cells in PPTC versus APTC tissue sections (Figure S5, Supporting Information). Our results revealed that ITGA2 fluorescence intensity in ITGA2^hi^‐PTC cells was significantly higher in PPTC samples compared to APTC samples (Figure 2g,h). This finding was corroborated in public datasets, where ITGA2 expression levels were significantly higher in PPTC samples compared to APTC samples (Figure 2i). Analysis of single‐cell sequencing data revealed significantly higher ITGA2 expression in BRAFV600E‐mutant PPTC cells compared to wild‐type cells (Figure 2j).

Furthermore, by analyzing the TCGA‐THCA dataset, we found that ITGA2 expression was significantly upregulated in tumor tissues and positively correlated with the malignant biological behavior of PTC (Figure 2k). Although ITGA2 expression did not exhibit a linear correlation with composite AJCC stages, its significant association with advanced T stages and nodal metastasis implicates ITGA2 in regulating invasive and metastatic phenotypes, distinct from overall stage progression.

#### 2.2.2 Activation of the Glycolysis Pathway in ITGA2^hi^‐PTC Cells Promotes the Proliferation of PPTC Cells

**Figure 3:**
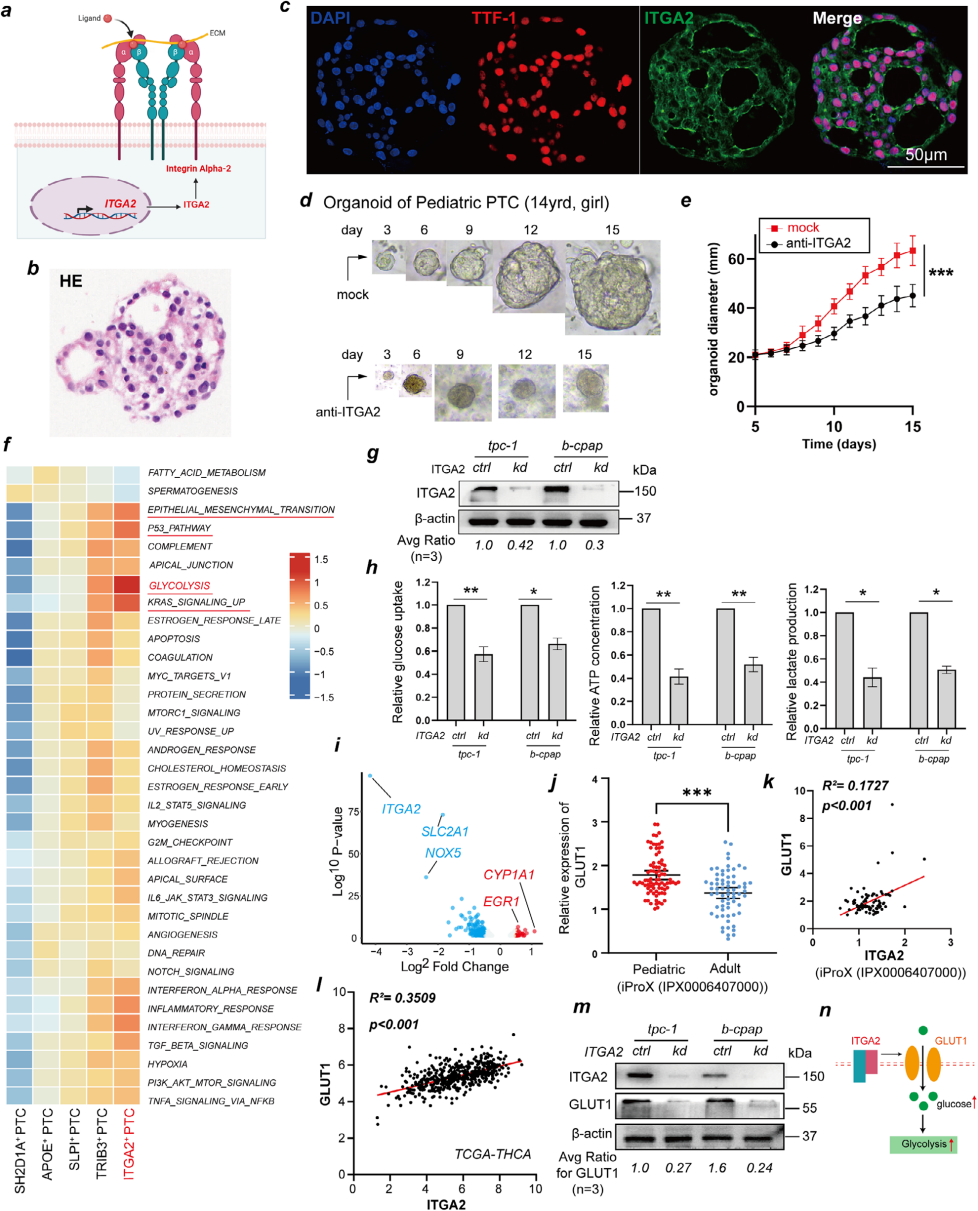
ITGA2 drives glycolytic metabolism to activate PTC proliferation. **a)** Schematic illustration of ITGA2 structure and cell membrane localization. **b,c)** Representative images of H&E and mIHC staining of pediatric PTC‐derived organoids showing thyroid‐specific marker (TTF‐1) and ITGA2. Scale bar = 50 µm. **d)** Brightfield microscopy images of the growth of a representative pediatric PTC‐derived organoid from day 3 to day 15 following treatment with or without anti‐ITGA2 antibody. **e)** Quantification of organoid diameter changes (*n* = 4). **f)** Heatmap showing metabolic pathway enrichment scores of all PTC subclusters analyzed by scMetabolism (Scale bar ranging from −1.5 to 1.5). **g,h)** PTC cells (tpc‐1, b‐cpap) were transfected with ITGA2 knockdown plasmid, representative immunoblot reveals the expression of ITGA2, glucose uptake, and the production of lactate and ATP were determined. **i)** b‐cpap cells expressing stable ITGA2 knockdown or control shRNA, RNA expression levels were analyzed by Bulk‐RNA‐Seq, Volcano plot showing differentially expressed genes in knockdown versus control cells (FDR < 0.05; |Log2(fold change) | > 1). Red and blue dots significantly upregulated and downregulated genes, respectively. **j)** GLUT1 expression in pediatric (*n* = 85) and adult (*n* = 66) PTC tissues, data from iProX (IPX0006407000). **k,l)** Scatter plots depicting the association between GLUT1 and ITGA2 expression in PTC tissues, data from iProX (IPX0006407000) and TCGA. **m)** Representative immunoblot showing expression of ITGA2 and GLUT1 in B‐CPAP cells expressing stable ITGA2 knockdown or control shRNA. **n)** Working model for the regulation of ITGA2 on glycolysis. The results are shown as the mean ± SEM of three independent experiments. Two‐way ANOVA with Sidak's post‐hoc test **e**), Two‐sided student's t test **h,j**), Pearson's correlation analysis **k,l**) was used to estimate statistical significance, ^*^ *p *< 0.05; ^**^ *p *< 0.01; ^***^ *p* < 0.001. mIHC, Multiplex Immunohistochemistry. H&E, Hematoxylin and Eosin. TTF‐1, thyroid transcription factor‐1. scMetabolism, Single‐Cell Metabolism. FDR, False discovery rate. Bulk‐RNA‐Seq, bulk RNA sequencing. iProX, Integrated Proteome Resources. TCGA, The Cancer Genome Atlas. SEM, standard error of the mean.

*ITGA2* encodes integrin subunit alpha 2, which forms a heterodimer with a beta subunit, functioning as a transmembrane receptor for collagens and related proteins (**Figure** **3**a). To investigate the functional role of ITGA2 in PPTC, we employed a patient‐derived PPTC organoid model. Organoids were successfully established from PTC tissue obtained from a 14‐year‐old girl (Figure 3b). On day 15, organoids were harvested and subjected to multiplex immunohistochemical (mIHC) staining for thyroid transcription factor‐1 (TTF‐1), a thyroid‐specific marker (Figure 3c). Treatment with an anti‐ITGA2 antibody significantly inhibited PPTC organoid growth (Figure 3d,e).

To investigate how ITGA2 promotes PPTC cell proliferation, we conducted functional enrichment analysis of each PTC cell subpopulation using gene set variation analysis (GSVA) at the single‐cell level. We found that ITGA2^hi^‐PTC cells were highly enriched in glycolysis, the KRAS signaling pathway, epithelial‒mesenchymal transition (EMT), oxidative stress, DNA repair, immune responses, inflammatory pathways, and viral response pathways (Figure 3f). We focused on how ITGA2 regulates glycolysis because our previous studies revealed that increased glycolysis levels promote the invasion and metastasis of PTC cells.^[^ ^15^ ^]^ Thus, we cultured two PTC cell lines (TPC‐1 and B‐CPAP) and successfully established stable ITGA2 knockdown models (Figure 3g). Consistent with the results of the scRNA‐seq data analysis, upon ITGA2 knockdown, glucose uptake and ATP and lactate production were significantly reduced in PTC cells (Figure 3h).

To elucidate the molecular mechanism by which ITGA2 regulates glycolysis, we performed bulk RNA sequencing on PTC cell lines with and without ITGA2 knockdown and found that SLC2A1 was significantly downregulated in knockdown cells (Figure 3i). Interestingly, SLC2A1 encodes GLUT1, a central regulator of cellular glucose uptake.^[^ ^12^ ^]^ We observed in the data published by Wang et al. that GLUT1 expression was significantly upregulated in PPTCs^[^ ^16^ ^]^ (Figure 3j) and confirmed a positive correlation between GLUT1 and ITGA2 expression (Figure 3k,l). Moreover, by western blotting, we confirmed that GLUT1 expression was significantly downregulated upon ITGA2 knockdown (Figure 3m). These results indicate that ITGA2 promotes glycolysis in PTC cells by increasing GLUT1 expression (Figure 3n). Although further evidence is required to fully elucidate how ITGA2 regulates GLUT1, we propose the SOX9‐ITGA2‐GLUT1 axis may orchestrate glucose transporter clustering via integrin‐mediated membrane partitioning, analogous to the β1‐integrin–MCT4/CD98 complex that coordinates proton‐coupled metabolite transport.^[^ ^17^, ^18^ ^]^

### 2.3 ITGA2^hi^‐PTC Cells Undergo a Metabolic Shift Toward Glycolysis Induced by the Key Transcription Factor SOX9

**Figure 4:**
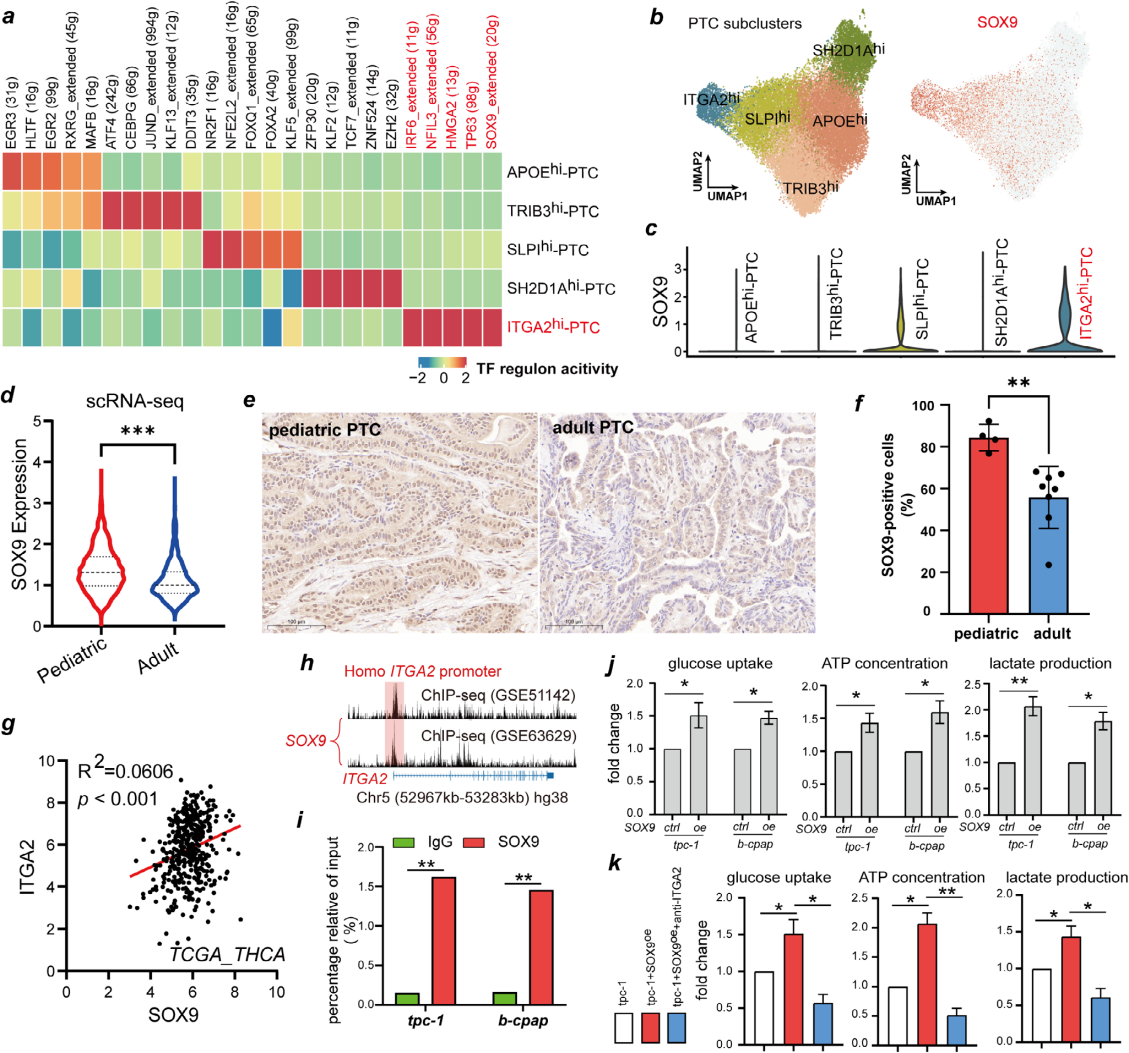
Glycolytic metabolism driven by ITGA2 is controlled by SOX9. **a)** Heatmap displaying the normalized activity of the top five transcription factor (TF) regulons predicted by the SCENIC algorithm across PTC cell subtypes. **b)** UMAP plot illustrating the distribution of PTC subpopulations (left panel); feature plots showing the expression of the transcription factor SOX9 (right panel), with cells expressing high levels of SOX9 highlighted in red. **c)** Violin plots depicting SOX9 expression in PTC cell subtypes. **d)** Violin plots showing the expression of SOX9 between pediatric PTC and adult PTC groups in our single‐cell RNA sequencing data. **e)** Representative immunohistochemical (IHC) images of SOX9 expression in pediatric and adult PTC tissues. Scale bar = 100 µm. **f)** Bar graph showing the proportion of SOX9‐positive cells in pediatric PTC (*n* = 4) and adult PTC (*n* = 9). **g)** Pearson correlation analysis of ITGA2 and SOX9 expression in the TCGA‐THCA cohort (*n* = 512). **h)** Prediction of SOX9 and ITGA2 promoter binding regions, data from GSE51142 and GSE63629 in the Cistrome DB database. **i)** ChIP‐qPCR was performed to verify the transcription of ITGA2 by SOX9. **j,k)** PTC cells (tpc‐1, b‐cpap) transfected with SOX9‐overexpressed plasmid, or co‐treated with anti‐ITGA2, glucose uptake and the production of lactate and ATP were determined. The results are shown as the mean ± SEM of three independent experiments, Two‐sided Wilcoxon rank‐sum test **d**), Two‐way ANOVA Sidak's post‐hoc test **i**), Two‐sided student's t test **f,j,k**), Pearson's correlation analysis **g**) was used to estimate statistical significance, ^*^ *p *< 0.05; ^**^ *p *< 0.01; ^***^ *p* < 0.001. SCENIC, Single‐Cell Regulatory Network Inference and Clustering. TF, Transcription Factor. IHC, Immunohistochemistry. ChIP‐qPCR, Chromatin Immunoprecipitation Quantitative PCR. TCGA‐THCA, The Cancer Genome Atlas‐Thyroid carcinoma. Cistrome DB, Cistrome Data Browser.

To investigate the reason for the upregulated expression of ITGA2 in PPTCs, we performed SCENIC transcription factor activity analysis on the 5 PTC cell clusters and found that each cluster had its own specifically upregulated transcription factors (**Figure** **4**a **;** Table S5, Supporting Information). Transcription factors (TFs) associated with tumor metastasis and progression, such as SOX9, HMGA2, TP63, NFIL3, and IRF6, exhibited high transcriptional activity in the ITGA2^hi^‐PTC subcluster (Figure 4a). SOX9 has been identified as a transcription factor related to sustained cancer stem cell traits, and its role in promoting tumor progression is well established.^[^ ^19^, ^20^ ^]^ UMAP visualization revealed that SOX9 expression in ITGA2^hi^‐PTC cells was significantly higher than that in other cell subpopulations (Figure 4b,c). By quantifying SOX9 expression in scRNA‐seq data, we found that SOX9 levels were significantly higher in PPTC cells than in APTC cells (Figure 4d). In addition, we observed the same phenomenon via IHC: the proportion of SOX9‐positive cells in PPTC tissue sections was significantly higher than that in APTC tissue sections (Figure 4e,f). Similarly, in the TCGA‐THCA dataset, SOX9 expression was positively correlated with ITGA2 expression (Figure 4g). Therefore, we hypothesized that SOX9 regulates ITGA2 expression.

To test this hypothesis, we performed validation experiments. Analysis of two ChIP‐Seq datasets (GSE51142 and GSE63629) revealed that SOX9 binds to the ITGA2 promoter region, as evidenced by a distinct binding peak (Figure 4h). This binding was confirmed by ChIP‐qPCR (Figure 4i). These results demonstrate that SOX9 transcriptionally regulates ITGA2. Subsequent detection of glycolysis levels further verified the functional consequence of this regulation. After SOX9 overexpression, glucose uptake and ATP and lactate production were significantly increased in PTC cells, whereas co‐treatment with anti‐ITGA2 antibody inhibited the glycolytic increase induced by SOX9 overexpression, suggesting that SOX9 regulates the glycolysis pathway in PTC cells via ITGA2 (Figure 4j,k).

### 2.4 PPTC‐Associated Nonblast Clusters are Enriched in M2 Macrophages

**Figure 5:**
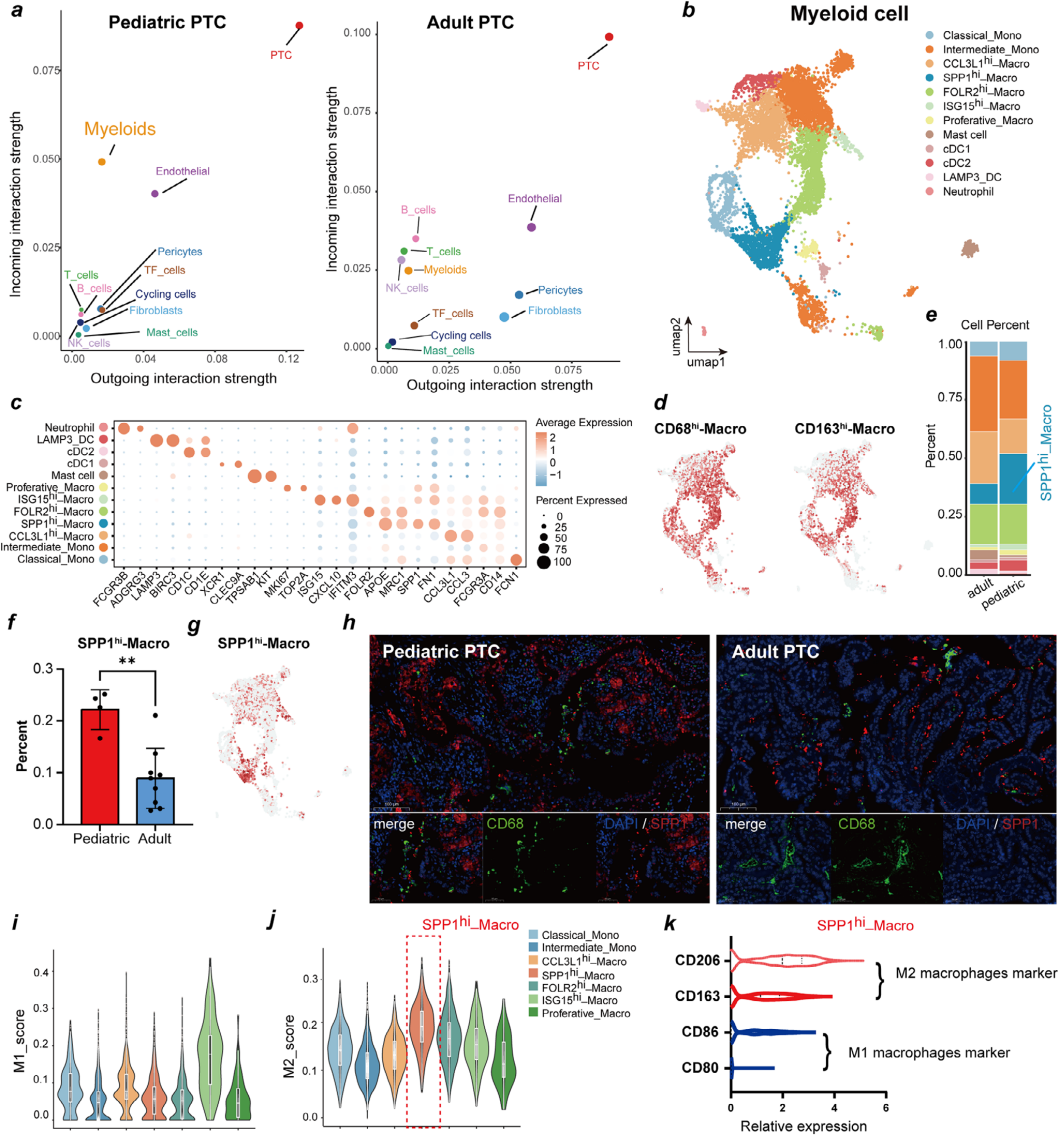
SPP1^hi^‐Macro represents a type of pediatric PTC‐associated macrophage. **a)** Bubble plot illustrates the cell communication strength between PTC and other cell types. **b)** UMAP plots show the composition of myeloid cells from pediatric and adult PTC samples. **c)** Dot plot show the expression of representative marker genes across each myeloid cell subset. **d)** Feature plots exhibiting the distribution of myeloid cells with high expression of CD68 and CD163. **e)** Bar chart demonstrating the proportion differences of myeloid cell subclusters between pediatric and adult PTC. **f)** Histogram illustrating the proportion of SPP1^hi^‐Macro cells in pediatric (*n* = 4) and adult PTC (*n* = 9). ^**^ *p *< 0.01, significance was determined using a two‐sided unpaired student's t‐test. Error bars represent the mean ± SD. **g)** Feature plots showing the distribution of myeloid cells with high expression of SPP1. **h)** Representative images of multiplex immunohistochemistry (mIHC) staining of SPP1^hi^‐Macro (positive for DAPI, CD68, SPP1) in pediatric and adult PTC. Scale bars, 100 and 50 µm. **i,j)** Violin plots depicting the scores of M1**i**)/M2**j**) polarization characteristics of macrophage cell subcluster. The median (horizontal line), first to third quartiles (box), and outer fences are being displayed. **k)** Violin plots illustrating the expression of M1 and M2 marker genes (CD206, CD163, CD80, CD86) in SPP1^hi^‐Macro. SPP1^hi^‐Macro, SPP1‐high Macrophage. DAPI, 4′,6‐diamidino‐2‐phenylindole. SD, Standard deviation. M1, classically activated macrophage. M2, alternatively activated macrophage.

A previous study revealed enhanced activation of the inflammatory and immune response in PPTC patients compared to APTC patients, which was associated with greater invasion and metastasis capabilities.^[^ ^16^ ^]^ Therefore, we further analyzed immune cell‐PTC cell interactions. We observed that overall cellular interaction frequency and intensity were greater in APTC than in PPTC, but the incoming signaling pathway score of myeloid cells in PPTCs was significantly higher than that in APTCs, suggesting enhanced interactions between myeloid cells and PTCs in PPTC (**Figure** **5**a; Figures S6,S7, Supporting Information). To systematically delineate immune microenvironment differences between PPTC and APTC, we observed the interaction between immune cells and PTC cells through cell communication analysis. Complementing this with comprehensive subcluster‐resolution characterization of key immune/stromal populations‐T/NK cells (Figure S8, Supporting Information), endothelial cells (Figure S9, Supporting Information), B cells (Figure S10, Supporting Information), and fibroblasts (Figure S11, Supporting Information)—we uncovered significantly attenuated interaction strength between PTC and non‐myeloid immune microenvironment constituents in PPTC. Myeloid cells constituted the dominant regulators of the PPTC immune microenvironment, supported by a significantly higher proportion of myeloid cells in PPTC versus APTC samples (Figure 1e), suggesting their involvement in regulating PPTC pathobiology.

We subsequently selected myeloid cells for further analysis and reclustered 10013 cells, identifying a total of 19 clusters and 12 cell types based on marker genes; these included macrophages (SPP1^hi^‐Macro, CCL3L1^hi^‐Macro, FOLR2^hi^‐Macro, ISG15^hi^‐Macro, and Proliferative‐Macro), monocytes (Classical‐Mono and Intermediate‐Mono), mast cells, neutrophils, and cDCs (cDC1, cDC2, and LAMP3_DC) (Figure 5b–d; Table S6, Supporting Information). SPP1^hi^‐Macro cells highly express SPP1 and APOE, and the proportion in PPTC samples was significantly more abundant in PPTC than in APTC samples (Figure 5e–g). SPP1 is secreted in various cancers, including PTCs, to drive invasion, metastasis, and therapy resistance.^[^ ^21^ ^]^ We observed that SPP1^hi^‐Macro engaged ligand‐receptor pairs (SPP1‐ITGA5/ITGB1/CD44) with PTC or endothelial cells to enhance communication (Figure S6, Supporting Information), suggesting a functional role in PPTC.

In clinical samples, the degree of SPP1^hi^‐Macro infiltration was higher in PPTC samples than in APTC samples by multiplex‐IHC (Figure 5h). Therefore, we calculated the monocyte‒macrophage M1/M2 polarization score to assess the functional role of SPP1^hi^‐Macro cells. We observed that most macrophage subtypes exhibited M2 polarization, with M2 scores exceeding M1 scores. Among them, SPP1^hi^‐Macro macrophages showed the highest M2 score, indicating their important role in promoting tumor progression and immunosuppression (Figure 5i–j). We further analyzed the expression of M1 macrophage markers (CD80 and CD86) and M2 macrophage markers (CD163 and CD206) in SPP1^hi^ macrophages. Consistent with these findings, the expression levels of M2 macrophage markers were significantly greater than those of M1 macrophage markers (Figure 5k). These results indicate that after interacting with PTC cells, macrophages specifically undergo M2 polarization.

### 2.5 ITGA2 Promotes Macrophage Polarization Toward the M2 Phenotype Through the JAK–STAT3 Signaling Pathway

**Figure 6:**
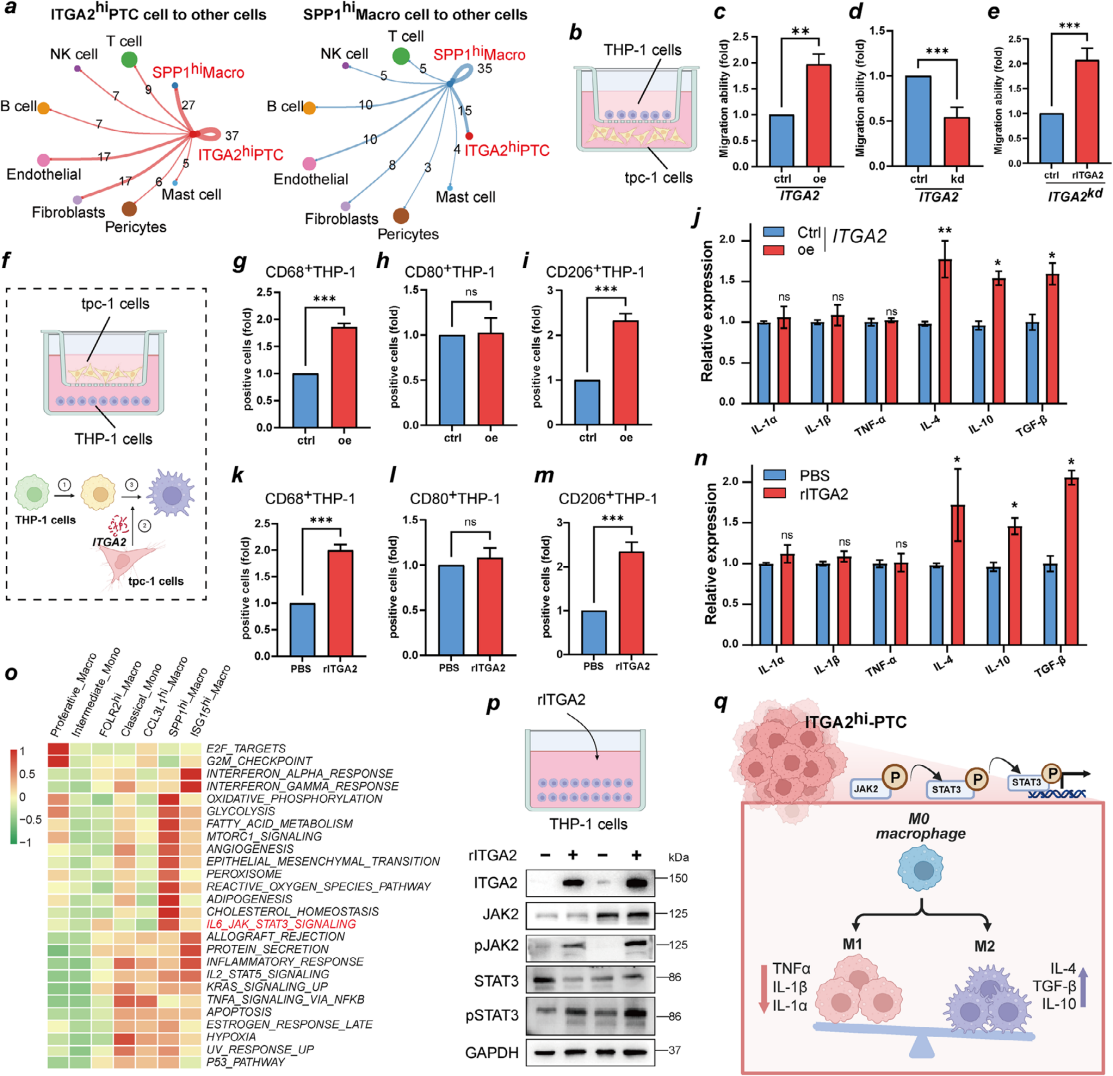
ITGA2^hi^‐PTC induces macrophage polarization. **a)** Circle plots show cell‐cell interaction strength differences of ITGA2^hi^‐PTC and SPP1^hi^‐Macro. **b)** Schematic diagram of in vitro chemotaxis of THP‐1 by ITGA2 via TPC‐1/THP‐1 conditioned media culture model. **c,d,e)** THP‐1 cells were differentiated by PMA pre‐treatment for 24 h, then cocultured with TPC‐1 cells for 2 days. Transwell assays were used to detect and observe the chemotaxis of THP‐1 after TPC‐1 cells were overexpressed‐ITGA2, knockdown‐ITGA2, or co‐treated with rITGA2. **f)** Schematic depiction of experimental design for macrophage phenotype polarization by ITGA2^hi^‐PTC, ① THP‐1 cells were stimulated with PMA to differentiate into M0,② PTC cells are allowed to generate ITGA2, ③ M0 macrophages are exposed to ITGA2. **g,h,i)** The expression levels of surface markers in M0‐differentiated THP‐1 cells exposed to TPC‐1 cells with overexpressed ITGA2 using flow cytometry. **j)** ELISA detected the level change of IL‐1α, IL‐1β, TNF‐α, IL‐4, IL‐10, and TGF‐β in THP‐1 cell supernatant after exposed to TPC‐1 cells with overexpressed ITGA2. **k,l,m)** The expression levels of surface markers in M0‐differentiated THP‐1 cells exposed to rITGA2 using flow cytometry. **n)** ELISA detected the level change of IL‐1α, IL‐1β, TNF‐α, IL‐4, IL‐10, and TGF‐β in THP‐1 cell supernatant after exposed to rITGA2. **o)** Heatmaps show the activity intensity of macrophage subtypes in selected hallmark pathways by GSVA analysis, Z‐score normalized: range −1–1. **p)** Representative immunoblot reveals the expression change of ITGA2, JAK2, STAT3, p‐JAK2, and p‐STAT3 in THP‐1 cells exposed to rITGA2. q)Schematic model depicting the mechanism by which ITGA2^hi^‐PTC cells drive M2 macrophage polarization via JAK2‐STAT3 signaling activation.​The results are shown as the mean ± SEM of three independent experiments. Two‐sided student's t test was used to estimate statistical significance, ^*^ *p *< 0.05; ^**^ *p *< 0.01; ^***^ *p* < 0.001; ns, no significance. ITGA2^hi^‐PTC, ITGA2‐high expressing papillary thyroid carcinoma cells. THP‐1, Human leukemia monocytic cell line. PMA, Phorbol 12‐myristate 13‐acetate. rITGA2, Recombinant ITGA2 protein. M0, Non‐ polarized macrophage. ELISA, Enzyme‐linked immunosorbent assay. GSVA, Gene set variation analysis. p‐JAK2, Phosphorylated JAK2. p‐STAT3, Phosphorylated STAT3.

The cell communication results revealed that ITGA2^hi^‐PTC and SPP1^hi^‐Macro cells had the strongest interaction, suggesting that ITGA2^hi^‐PTC cells are involved in regulating macrophage polarization (**Figure** **6**a). We further analyzed whether conditioned medium from PTC cells had a chemotactic effect on macrophages (THP‐1) (Figure 6b). Conditioned medium from ITGA2^oe^‐TPC‐1 cells exerted significantly greater chemotactic effect on THP‐1 cells than that from control cells in a Boyden chamber assay (Figure 6c). However, conditioned medium from ITGA2^kd^‐TPC‐1 cells had the opposite effect (Figure 6d). The direct effect of ITGA2 on monocyte migration and polarization was also evaluated by adding recombinant ITGA2 (rITGA2) to THP‐1 cells. The results demonstrated that rITGA2 directly promotes the migration of THP‐1 cell (Figure 6e). Flow cytometry showed that conditioned medium from ITGA2^oe^‐TPC‐1 cells preferentially induced monocyte differentiation into M2 macrophages (CD206+) over M1 macrophages (CD80+) (Figure 6f–i). Similarly, levels of M2 macrophage‐associated cytokines (*IL‐4, IL‐10, TGF‐β)* were significantly higher in THP‐1 cells cocultured with ITGA2^oe^‐TPC‐1 cells than in those cocultured with control cells (Figure 6j). In contrast, M1 macrophage‐associated cytokines (*IL‐1α*, *IL‐1β*, *TNF‐α)* were downregulated or unchanged in THP‐1 cells cocultured with ITGA2^oe^‐TPC‐1 cells (Figure 6j). In fact, adding rITGA2 to THP‐1 cell culture medium also led to results consistent with those of ITGA2^oe^‐TPC‐1 coculture (Figure 6k–n). These results established a dose‐dependent relationship between ITGA2 and macrophage polarization: ITGA2^hi^‐PTC induces M2 macrophage polarization by secreting ITGA2.

We investigated mechanisms underlying ITGA2‐mediated M2 polarization. By scoring the functional enrichment of the macrophage subpopulations, we found that SPP1^hi^‐Macro cells were significantly enriched in IL6_JAK_STAT3_SIGNALING, EMT, glycolysis, and other pathways (Figure 6o). Since JAK–STAT3 activation promotes macrophage recruitment, M2 polarization, and immunosuppression during tumor progression,^[^ ^22^ ^]^ we hypothesized that ITGA2^hi^‐PTC regulates M2 polarization via JAK2/STAT3 signaling. We also used a coculture system for verification, and the western blot results revealed that overexpression of ITGA2 promoted the expression of pJAK2 and pSTAT3 (Figure 6p). These findings confirmed that ITGA2^hi^‐PTC cells promote the M2 polarization of macrophages by activating the JAK–STAT3 signaling pathway (Figure 6q).

## 3 Discussion

The sustained annual increase in pediatric papillary thyroid carcinoma (PPTC) incidence and its distinct biological manifestations compared to adult PTC (APTC) have garnered significant scientific interest.^[^ ^23^, ^24^, ^25^ ^]^ Single‐cell RNA sequencing (scRNA‐seq) has emerged as a transformative methodology for delineating APTC tumor ecosystems, identifying critical cellular constituents driving oncogenesis^[^ ^13^, ^14^ ^]^ and elucidating mechanisms underlying spatial heterogeneity and metastatic progression.^[^ ^26^, ^27^ ^]^ Given the established clinicopathological and molecular disparities between APTC and PPTC, coupled with historical research emphasis on adult populations, systematic investigation of pediatric‐specific tumor biology through cellular‐resolution profiling becomes imperative. To our knowledge, this study provides the first single‐cell transcriptomic atlas of ITGA2^hi^‐PTC cells in pediatric cohorts, delineating preliminary but mechanistically instructive insights into the distinct biological programs underlying PPTC pathogenesis.

Our multimodal analysis revealed substantial interpatient heterogeneity among PTC cellular subpopulations, with PPTC specimens exhibiting marked enrichment of ITGA2^hi^‐PTC cells. This distinct subpopulation demonstrated elevated expression of ITGA2, KRT6A, SOSTDC1, KRT6B, and KRT14 —all molecular markers extensively characterized in epithelial‐mesenchymal transition and tumor progression.^[^ ^28^, ^29^, ^30^, ^31^ ^]^ Pseudotemporal trajectory reconstruction and stemness quantification further positioned ITGA2^hi^‐PTC cells as progenitor‐like entities within differentiation hierarchies, characterized by a high dedifferentiation state (low TDS/high CytoTRACE). Notably, developmentally adjacent SLPI^hi^‐PTC subclusters exhibited paradoxical dedifferentiation signatures and distinct metabolic profiles. This biological divergence arises through two synergistic mechanisms: 1) Active reprogramming into a hybrid cellular state co‐expressing partial epithelial redifferentiation markers (KRT8/19/MUC1) alongside progenitor‐associated traits; 2) Inherent limitations of differentiation scoring systems, where pathway‐specific markers enriched in SLPI^hi^‐PTC subpopulations systematically bias transitional state classification. These findings collectively indicate that ITGA2 overexpression not only drives proliferative capacity but also regulates non‐linear phenotypic transitions underlying pediatric tumor aggressiveness.

Functionally, ITGA2 (integrin subunit α2) orchestrates a dysregulated signaling axis through β1‐integrin heterodimerization (α2β1), governing bidirectional extracellular matrix crosstalk that modulates neoplastic adhesion, metastatic motility, cancer stemness maintenance, and tumor angiogenesis.^[^ ^32^ ^]^ While prior investigations established ITGA2 overexpression as an independent prognostic determinant in APTC,^[^ ^33^, ^34^ ^]^ its pathogenetic role in pediatric thyroid carcinogenesis remained unresolved. Our integrated multi‐cohort analysis revealed consistent and significant ITGA2 upregulation in PPTC relative to APTC. Notably, external bulk RNA‐seq data (GSE275666) corroborated this transcriptional divergence, though future studies warrant matched multi‐omics profiling to resolve temporal dynamics. Critically, comprehensive spatial quantification via immunofluorescence confirmed microenvironmental enrichment of ITGA2^hi^ tumor cells in pediatric cohorts, with 100% sample coverage ensuring biomarker verification robustness despite technical constraints precluding universal bulk RNA‐seq validation. Functional validation using antibody blockade in patient‐derived organoid models recapitulated ITGA2's tumor‐promotive effects, though conserved oncogenic mechanisms between pediatric and adult cohorts warrant further exploration. Notably, preliminary mechanistic investigations revealed ITGA2‐mediated GLUT1 upregulation, potentially linking integrin signaling to metabolic reprogramming in PPTC. This observation aligns with established connections between subcellular integrin localization and functional modulation, as exemplified by miR‐99a‐3p‐induced ITGA2 internalization inhibiting PTC metastasis.^[^ ^33^ ^]^ Further elucidation is required to clarify that although EMT signatures were also enriched in ITGA2^hi^‐PTC subclusters, glycolytic drivers demonstrated a stronger association with malignant phenotypes. Future research should aim to resolve the crosstalk between EMT and metabolism in the progression of PTC.

The tumor microenvironment (TME) analysis uncovered ITGA2's emerging role as a mediator of immune‐stromal crosstalk. Our data demonstrate ITGA2^hi^‐PTC cell‐driven polarization of tumor‐associated macrophages (TAMs) toward immunosuppressive M2 phenotypes via JAK2/STAT3 activation. Spatial characterization identified SPP1^hi^‐Macro cell enrichment in PPTC microenvironments ‐ a myeloid subpopulation associated with therapeutic resistance in multiple malignancies.^[^ ^35^, ^36^ ^]^ These M2‐polarized macrophages secreted protumorigenic cytokines (IL‐4, TGF‐β, IL‐10), establishing a feedforward loop that amplifies metastatic potential.^[^ ^37^, ^38^, ^39^ ^]^ This mechanistic framework aligns with prior observations of BRAF^V600E^‐mediated myeloid recruitment in thyroid cancer progression,^[^ ^40^ ^]^ suggesting convergent pathways in pediatric and adult disease evolution.

Several methodological limitations merit consideration. First, inherent patient heterogeneity and cohort size constraints necessitated rigorous validation through internal‐external dataset triangulation. Second, translational investigations were restricted to organoid models due to current limitations in pediatric thyroid cancer xenograft systems. Third, while bioinformatic deconvolution and in vitro assays elucidated ITGA2‐mediated TME remodeling, direct functional evidence of macrophage‐driven metastasis requires further experimental validation.

## 4 Conclusion

**Figure 7:**
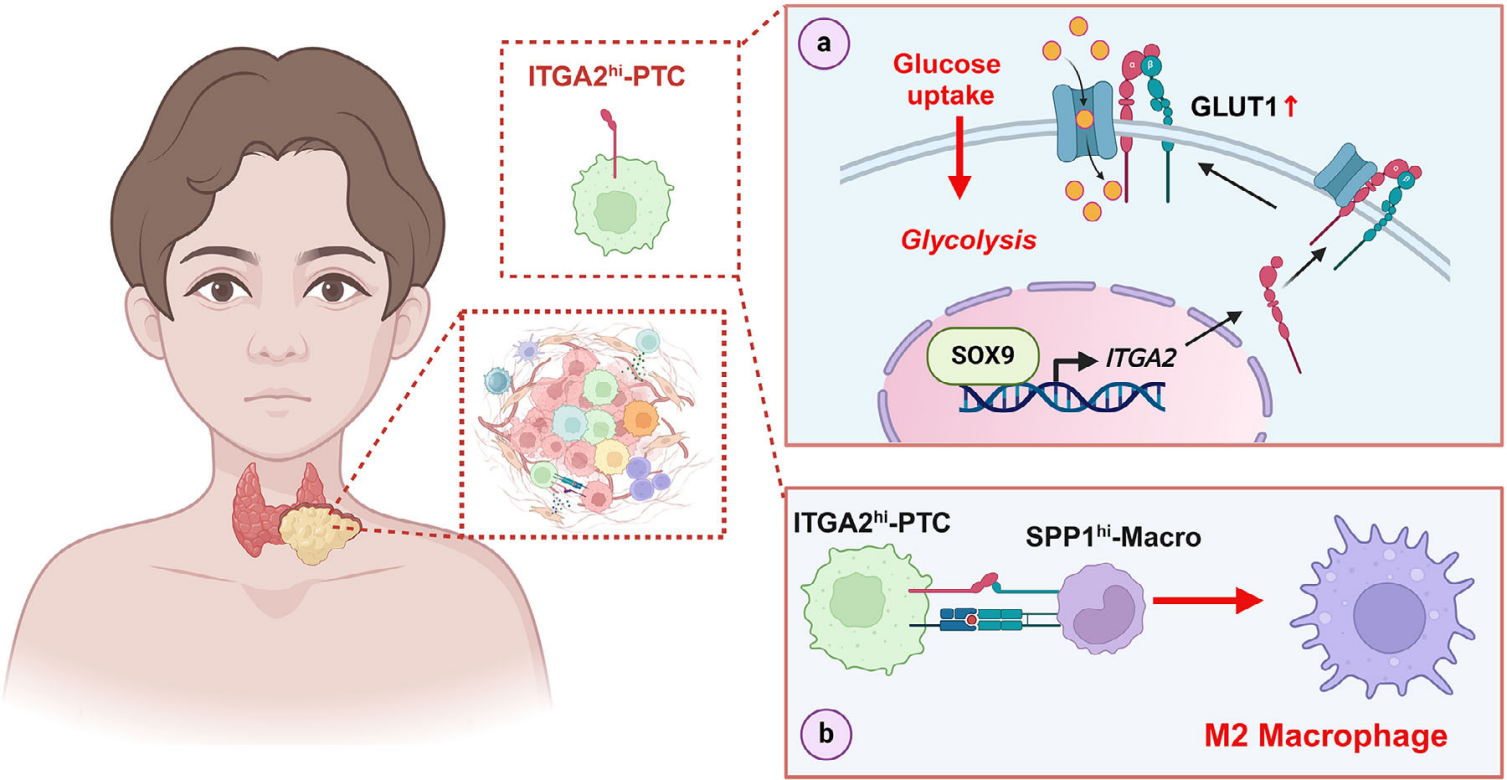
Schematic Diagram of the present study. Schematic Diagram Dissecting ITGA2^hi‐^PTC cells regulating glycolysis and the immune microenvironment in pediatric PTC. Image created with BioRender.com.

This multiscale investigation establishes ITGA2^hi^‐PTC cells as central orchestrators of PPTC progression through dual mechanisms: metabolic reprogramming via GLUT1 modulation and immune microenvironment remodeling through M2 macrophage polarization (**Figure** **7**). These findings provide a conceptual framework for developing targeted therapies against PPTC's distinct pathogenic drivers, particularly for patients with advanced or recurrent disease.

## 5 Experimental Section

### Patient Cohort and Specimen Processing

This study included 13 papillary thyroid carcinoma (PTC) patients who underwent surgery at Xiangya Hospital, Central South University, from January 2023 to June 2024. The cohort was divided into four pediatric PTC (PPTC) cases (under 18) and nine adult PTC (APTC) cases (18 and older). Diagnosis was confirmed through intraoperative frozen section cytology and postoperative examination of paraffin‐embedded tissues. Patients with a history of preoperative antitumor therapies were excluded. Supplementary Table S1 provides detailed demographic and clinicopathological data. The study was approved by the Xiangya Hospital Institutional Review Board (Protocol No. 202403837), and informed consent was obtained from all participants or their legal guardians.

### Preparation of Single‐Cell Suspensions From Papillary Thyroid Carcinoma Specimens

Freshly resected tumor specimens were immediately placed in GEXSCOPE tissue preservation solution (Singleron Biotechnologies) for stabilization. They were then washed three times with Hank's balanced salt solution (HBSS) to remove the preservation medium. The tissues were cut into 1–2 mm^3^ pieces with sterile blades and enzymatically digested in 2 mL of GEXSCOPE Tissue Dissociation Solution at 37 °C for 15 min with constant agitation. The cell suspension was filtered through a 40 µm cell strainer to eliminate undigested tissue, then centrifuged at 300 × g for 5 min at 4 °C. The pelleted cells were resuspended in 1 mL of filter‐sterilized PBS and assessed for viability (>85% via trypan blue exclusion) and concentration using a hemocytometer under phase‐contrast microscopy.

### Single‐Cell RNA Sequencing

Single‐cell RNA sequencing was performed by loading viable single‐cell suspensions (1 × 10⁵ cells mL^−1^ in PBS) onto a GEXSCOPE microfluidic system for cell partitioning. Libraries were created using Singleron Biotechnologies' protocol, adding unique molecular identifiers (UMIs) and cell barcodes during reverse transcription. The scRNA‐seq libraries were then sequenced on an Illumina HiSeq X10 with 150 bp paired‐end reads.

### ScRNA‐Seq Data Processing and Cell Type Annotation

Raw sequencing reads from Singleron GEXSCOPE scRNA‐seq experiments were processed into gene expression matrices. Initially, fastQC and fastp were used for quality control, removing reads below Q30. Cutadapt trimmed adapter sequences and poly‐A tails, keeping reads over 20 bp. Cell barcodes and UMIs were extracted, and reads were aligned to the GRCh38 genome with STAR. Gene expression and UMI counts were quantified using FeatureCounts. Count matrices were imported into Seurat (version 5.1.0) for quality control, filtering out cells with fewer than 200 genes or over 15% mitochondrial content.^[^ ^41^ ^]^ Doublets were identified with DoubletFinder (version 2.0.4) at a 5% rate.^[^ ^42^ ^]^ Normalization and scaling addressed UMI count variability. Batch correction was performed using Harmony (version 1.2.3) with default settings.^[^ ^43^ ^]^ After thorough quality filtering, 90234 high‐confidence single cells were kept for further analysis. Dimensionality reduction was achieved through PCA on 2000 highly variable genes, followed by UMAP visualization (resolution = 0.8) using Seurat's FindNeighbors and FindClusters. Cell types were annotated using marker genes from CellMarker (version 2.0) and PanglaoDB (2022 update).^[^ ^44^, ^45^ ^]^

### Pseudotemporal Trajectory Reconstruction

The developmental trajectories were deduced using Monocle3 (version 1.3.7) through a structured workflow as follows: Seurat objects were converted into the CellDataSet format using the new_cell_data_set function.^[^ ^46^ ^]^ Dimensionality reduction was performed using the reduce_dimension method, which was subsequently followed by trajectory inference conducted via the order_cells algorithm. The potential for cellular differentiation was quantitatively evaluated using CytoTRACE2 (version 1.0.0), wherein higher CytoTRACE scores indicate an increased level of stemness.^[^ ^47^ ^]^

### Transcriptional Regulatory Network Analysis

To identify transcription factor regulons, SCENIC (version 1.1.3) was employed.^[^ ^48^ ^]^ Coexpression networks were constructed using GRNBoost2, followed by cis‐regulatory motif analysis conducted with RcisTarget. Regulon activity scores for individual cells were calculated using AUCell. Differential regulon activity among subtypes was assessed using Wilcoxon rank‐sum tests, applying a minimum percentage threshold of 0.2 (min.pct = 0.2) and a log_2_‐fold change threshold of 0.25 (log_2_FC threshold = 0.25).

### Intercellular Communication Mapping

The profiling of ligand‐receptor interactions was systematically performed utilizing CellChat (version 1.5.0) in conjunction with the CellChatDB.human database (version 1.0).^[^ ^49^ ^]^ Probabilistic communication networks were computed using the computeCommunProb function, while pathway‐level inferences were obtained through the application of the computeCommunProbPathway function. The visualization of signaling hierarchies was accomplished through the implementation of circular layouts and heatmaps.

### Functional Enrichment Analysis

Gene Ontology (GO) and Kyoto Encyclopedia of Genes and Genomes (KEGG) pathway analyses were performed utilizing the clusterProfiler package (version 4.0.5).^[^ ^50^ ^]^ The significance of enrichment was determined by a false discovery rate (FDR) threshold of less than 0.05, as adjusted by the Benjamini‐Hochberg procedure.

### Differential Expression Profiling

Differentially expressed genes (DEGs) were discerned employing Seurat's FindMarkers function, applying the Wilcoxon test with criteria of a minimum percentage (min.pct) of 0.2, a log_2_ fold change (log_2_FC) exceeding 1, and an adjusted *p*‐value less than 0.05. For bulk RNA‐seq analyses, DESeq2 (version 1.38.3) was utilized for processing count data, while limma (version 3.54.2) was employed for microarray normalization.^[^ ^51^, ^52^ ^]^

### Phenotypic Signature Quantification

The gene signature scores, notably the Thyroid Differentiation Score (TDS) and the M1/M2 macrophage polarization scores, were computed using the AUCell (version 1.20.2) and Gene Set Variation Analysis (GSVA, version 1.52.3) tools, with the assistance of published gene panels.^[^ ^53^ ^]^ Comprehensive details pertaining to the gene signatures are furnished in Table S7 (Supporting Information).

### Integration of TCGA and GEO Data

The TCGA‐THCA RNA‐seq data (IlluminaHiSeq_RNASeqV2) were acquired from the UCSC Xena portal (release version: 2023‐05). The GEO datasets, namely GSE275666, GSE51142, and GSE63629, were processed utilizing GEOquery (version 2.68.2). The scRNA‐seq data for verification were obtained from the GEO database (GSE281736), and the analysis method was consistent with that of the sequencing data. The proteomic data were retrieved from iProX (IPX0006407000). Chromatin immunoprecipitation peaks were visualized within the Integrative Genomics Viewer (IGV, version 2.12.3).^[^ ^54^ ^]^

### Immunohistochemical Staining

Formalin‐fixed paraffin‐embedded (FFPE) tissue sections underwent antigen retrieval in citrate buffer (pH 6.0) at 95 °C for 15 min. Endogenous peroxidase activity was blocked with 0.3% hydrogen peroxide. The sections were then incubated overnight at 4 °C with a monoclonal anti‐SOX9 antibody (1:200 dilution; Bioss). For signal development, HRP‐conjugated secondary antibodies and 3,3′‐diaminobenzidine (DAB) were used. High‐resolution images of the stained sections were captured using a 3DHISTECH Pannoramic 250 scanner.

### Multiplex Immunofluorescence

A Multiplex Immunofluorescence Assay using Opal 7‐plex staining (PerkinElmer) was performed on FFPE tissue sections following the manufacturer's protocols. The antibody panel included CK7 (1:1000), ITGA2 (1:100), SPP1 (1:200), and CD68 (1:1000). Multispectral imaging was conducted with a PhenoImager HT (Akoya Biosciences), using DAPI for counterstaining.

### Cell Lines

The human PTC cell lines tpc‐1 and b‐cpap, and THP‐1 were obtained from Xiangya Hospital's Institute of Medical Sciences. The tpc‐1 and b‐cpap cells were cultured in DMEM with 10% FBS, penicillin, and streptomycin, they were kept at 37 °C with 5% CO2. For ITGA2 and SOX9 gene manipulation, cells were grown in complete medium for 24 h, then transfected with vectors from GeneChem using Lipofectamine 2000, following the manufacturer's instructions. The THP‐1 cells were cultured in RPMI‐1640 medium with 10% FBS at 37 °C and 5% CO2. They were differentiated into macrophages using 10 ng mL^−1^ phorbol 12‐myristate 13‐acetate (PMA) for 24 h before experiments.

### Patient‐Derived Organoid Culture

Freshly resected specimens were transported in DMEM/F12 + 1% penicillin‐streptomycin‐amphotericin B (P/S/A) on ice, washed with balanced salt solution (BSS) containing 1% P/S/A, and enzymatically dissociated in BSS with 400 U/mL collagenase I (Gibco 17100017), 400 U mL^−1^ collagenase II (Gibco 17101015), and 10 U/mL DNase I (37 °C, 30–60 min, agitation). Digestion was terminated with DMEM/F12 (Gibco 12634010) + 10% BSA, filtered through 100 µm strainers, and centrifuged at 1500 rpm (300 × g) for 5 min. After two washes with DMEM/F12, pelleted cells were resuspended in DMEM/F12, mixed with 3× volume Matrigel (Corning 356231), and plated as 50 µL droplets in pre‐warmed 24‐well plates. Following polymerization (37 °C, 10–30 min), cultures were overlaid with growth medium containing Wnt3a (50 ng mL^−1^, PeproTech 315‐20), R‐spondin‐1 (500 ng mL^−1^, PeproTech 120‐38), FGF‐10 (100 ng/ mL^−1^, PeproTech 100‐26), EGF (50 ng mL^−1^, PeproTech AF‐100‐15), Noggin (100 ng mL^−1^, PeproTech 120‐10C), Y‐27632 (10 µm, Sigma Y0503), N‐Acetyl‐L‐cysteine (1.25 mm, Sigma A9165), A83‐01 (0.5 µmol L^−1^, Sigma SML0788), GlutaMAX (1×, Gibco 35050061), B27 (1×, Gibco 17504044), and HEPES (10 mmol L^−1^, Gibco 15630106), refreshed every 3–7 days. For passaging, organoids were dissociated in TrypLE Express (37 °C, 5 min), quenched with Advanced DMEM/F12 + 10% BSA, centrifuged (1500 rpm, 5 min), and replated at 1:2–1:4 split ratios every 14 days.

### Western Blot

Cells were lysed utilizing RIPA buffer (Thermo Fisher Scientific, USA) supplemented with a protease/phosphatase inhibitor cocktail to facilitate the extraction of total protein. The extracted proteins were subsequently separated by SDS‐PAGE and transferred onto polyvinylidene fluoride (PVDF) membranes. Post‐transfer, the membranes were blocked with a blocking solution consisting of 5% bovine serum albumin (BSA) and 5% milk in Tris‐buffered saline with Tween 20 (TBST) at ambient temperature for a duration of 2 h. The specific primary antibodies employed included: β‐actin (Abcam, ab8227, dilution 1:5000), ITGA2 (Abcam, ab133557, dilution 1:10000), GLUT1 (Proteintech, 829‐1‐AP, dilution 1:4000), JAK2 (Proteintech, 17670‐1‐AP, dilution 1:600), pJAK2 (Cell Signaling Technology, 4406S, dilution 1:1000), STAT3 (Cell Signaling Technology, 9139S, dilution 1:1000), pSTAT3 (Cell Signaling Technology, 9145S, dilution 1:2000), and GAPDH (Proteintech, HRP‐60004, dilution 1:10000). Subsequently, secondary antibodies (goat anti‐mouse IgG, ShareBio, dilution 1:5000; goat anti‐rabbit IgG, ShareBio, dilution 1:5000) were incubated at room temperature for 2 h. The blots were visualized using an enhanced chemiluminescence detection kit (GE Healthcare, Buckinghamshire, UK), and all bands were analyzed using ImageJ software (version 1.8.0).

### Glucose Uptake, Lactate, and ATP Assay

The Glucose Uptake, Lactate Assay Kit II, and ATP Colorimetric Assay kits from Biovision were used to measure glucose uptake, lactate, and ATP production. For glucose uptake, cells (1 × 10^6^ per well) were plated in a 96‐well plate, glucose‐starved with 100 µL KRPH buffer with 2% BSA for 40 min, then treated with 10 µL of 10 mm 2‐DG for 20 min. For lactate and ATP assays, 1 × 10^6^ cells were homogenized in 100 µL of the kit's assay buffer, centrifuged, and the supernatant was tested. For lactate dehydrogenase activity, cells were lysed in 100 µL assay buffer, centrifuged at 10000 × g for 15 min at 4 °C, and 30 µL of supernatant was used in a 96‐well plate.

### ELISA

Enzyme‐Linked Immunosorbent Assay (ELISA) was employed to analyze cytokine levels in cell culture supernatants, which were meticulously collected for this purpose. The quantitative determination of cytokine concentrations was conducted using specific ELISA kits, adhering strictly to the detailed protocols provided by the respective manufacturers. For this study, Human IL‐1α, IL‐1β, TNF‐α, IL‐4, IL‐10, and TGF‐β ELISA Kits were sourced from Servicebio (Wuhan, China), ensuring the precise and reliable measurement of the targeted cytokines.

### Chromatin Immunoprecipitation (ChIP)‐qPCR

Chromatin immunoprecipitation coupled with quantitative PCR (ChIP‐qPCR) was employed to validate SOX9 binding to the ITGA2 promoter region, previously identified through ChIP‐seq peak analysis. Approximately 1×10^7^ tpc‐1 or b‐cpap cells were fixed with 1% formaldehyde at room temperature for 10 min, and the reaction was quenched with glycine. Cells were washed twice with ice‐cold PBS before chromatin fragmentation by sonication to 200–500 bp; 2% of chromatin was reserved as an input control. Immunoprecipitation was performed overnight at 4 °C using anti‐SOX9 antibody (MERCK, AB5535), with IgG serving as a non‐specific negative control. Immune complexes were captured using Protein A/G‐conjugated beads and sequentially washed. Following crosslink reversal and DNA purification, qPCR amplification utilized SYBR Green‐based detection with primers targeting the ChIP‐seq peak region, and fold enrichment was quantified by the ΔΔCt method. Binding was considered significant if enrichment exceeded IgG levels by ≥3‐fold.

### Polarization of Differentiated THP‐1

THP‐1 cells were cultured at a concentration of 5 × 10^6^ cells mL^−1^ in RPMI medium (Gibco), supplemented with 10% heat‐inactivated fetal bovine serum (FBS; HyClone, GE Healthcare Life Sciences) and 1% penicillin/streptomycin (Sigma‐Aldrich). Differentiation of THP‐1 cells into M0 macrophages was achieved through treatment with PMA at a concentration of 100 ng/mL for 48 h. Subsequently, M0‐differentiated THP‐1 cells (1 × 10^6^ cells) were exposed to TPC‐1 cells that were either overexpressing ITGA2, subjected to ITGA2 knockdown, or co‐treated with recombinant ITGA2 (rITGA2). To assess macrophage phenotype polarization, the polarized THP‐1 cells were detached using trypsin supplemented with EDTA (Life Technologies), fixed with Phosflow buffer (BD Biosciences), and stained with fluorescence‐labeled anti‐human monoclonal antibodies: CD68 (PerCP, BD Biosciences), CD80 (PE, BioLegend), and CD206 (FITC, BD Biosciences). Flow cytometric analysis was conducted using a BD FACSCalibur flow cytometer, and data were analyzed with FlowJo software.

### Statistical Analysis

Analyses were performed using R (version 4.3.1) and GraphPad Prism (version 9.5.0). Data are shown as means ± SEM from at least three independent experiments. Normality was assessed using the Shapiro‐Wilk test (*α* = 0.05). Statistical significance was determined by: i) two‐tailed unpaired Student's *t*‐test (normal distribution) or Wilcoxon rank‐sum test (non‐normal distribution); ii) one‐way/two‐way ANOVA with Tukey's/Sidak's post hoc test (parametric multi‐group comparisons); iii) Kruskal–Wallis test with Dunn's post hoc correction (non‐parametric multi‐group analyses). All tests were two‐sided with *α* = 0.05. Significance thresholds were set at ^*^ *p* < 0.05, ^**^ *p* < 0.01, ^***^ *p* < 0.001.

### Ethics Approval and Consent to Participate

The study was approved by the Ethics Committee of Xiangya Hospital, Central South University (Protocol No. 202403837). Informed consent was obtained from all individual participants included in the study.

## Conflict of Interest

The authors declare no conflict of interest.

## Author Contributions

Z.Z.J., N.L. and Y.S. performed methodology; N.L. and Y.S., L.C., J.D.Y., Y.H.S., J.X.Z., Z.C.L., H.L.T. and N.T. contributed to experiment; Z.Z.J., N.L. and Y.S. wrote the original draft; S.C., P.H. wrote‐reviewed and edited the final manuscript; L.C., J.D.Y. and Y.H.S. contributed to data curation; J.X.Z. supervised the project; P.H., S.C. and N.T acquired funding. All authors read and approved the final manuscript. Z.Z.J., N.L., and Y.S. contributed equally to this work.

## Supporting information



Supporting Information

Supporting Information

## Data Availability

The data that support the findings of this study are available from the corresponding author upon reasonable request.
