# Supplementary material for: Single‐Cell Transcriptomics Reveals ITGA2‐Mediated Metabolic Reprogramming and Immune Crosstalk in Pediatric Thyroid Carcinogenesis
Source: Adv Sci (Weinh). 2025 Sep 23;12(45):e04088. doi: 10.1002/advs.202504088 (PMC12677704; doi:10.1002/advs.202504088)
Supplement: Supplementary file 1 — Supporting Information [file ADVS-12-e04088-s002.docx]

Supporting Information

Single-Cell Transcriptomics Reveals ITGA2-Mediated Metabolic Reprogramming and Immune Crosstalk in Pediatric Thyroid Carcinogenesis

Zhi-jun Zhan^#^, Ning Li^#^, Yan Sun^#^, Lu Chen, Jun-da Yin, Yun-han Shan, Jia-xin Zeng, Zhe-chen Li, Hai-long Tan, Neng Tang, Shi Chang and Peng Huang^*^

**Contents for Supporting Information**

**S1. List of Supplementary tables**

**S2. List of Supplementary figures**

**S3. Additional Data**

**S3.1 Expansion and Supplementary Content**

S3.1.1 Microscopic morphologies of PTC samples.

S3.1.2 Single-cell expression profiling of pediatric and adult PTC.

S3.1.3 Validate the expression of ITGA2^hi^-PTC cell marker genes in GEO data.

S3.1.4 Characteristics of heterogeneous cancer cell subtypes in thyroid cancer.

S3.1.5 mIHC staining of ITGA2^hi^-PTC cells in PTC samples.

S3.1.6 Intercellular communication in PPTC and APTC revealed by CellChat.

**S3.2 Supplementary Analysis**

S3.2.1 Heterogeneity Landscape of T/NK Cells between PPTC and APTC

S3.2.2 Heterogeneity Landscape of endothelial cells between PPTC and APTC

S3.2.3 Heterogeneity Landscape of B cells between PPTC and APTC

S3.2.4 Heterogeneity Landscape of stromal cells between PPTC and APTC

**S1. List of Supplementary tables**

Supplementary table 1. Samples information related to Figure 1

Supplementary table 2. List of signature genes of PTC cell clusters, related to Figure 2

Supplementary table 3. Enriched Pathways Corresponding to Subtype-Specific Expressed Genes in PTC cell, related to Figure 2

Supplementary table 4. TDS score & Cytotrace score of PTC cell，related to Figure 2

Supplementary table 5. Top 5 transcription factors of PTC cells, related to Figure 4

Supplementary table 6. List of signature genes of myeloid cell clusters, related to Figure 5

Supplementary table 7. List of gene signature

**S2. Supplementary figures list:**

Supplementary figure 1. Microscopic morphologies of PTC samples.

Supplementary figure 2. Single-cell expression profiling of pediatric and adult PTC.

Supplementary figure 3. Validate the expression of ITGA2^hi^-PTC cell marker genes in GEO data.

Supplementary figure 4. Characteristics of heterogeneous cancer cell subtypes in thyroid cancer.

Supplementary figure 5. mIHC staining of ITGA2^hi^-PTC cells in PTC samples.

Supplementary figure 6. Intercellular communication in PPTC and APTC revealed by CellChat.

Supplementary figure 7. mIHC staining reveals spatial co-localization of SPP1^hi^-macrophages and ITGA1^hi^-PTC cells in PTC samples.

Supplementary figure 8. T and NK cell subtypes in PPTC and APTC.

Supplementary figure 9. Endothelial cell subtypes in PPTC and APTC.

Supplementary figure 10. B cell subtypes in PPTC and APTC.

Supplementary figure 11. Pericyte/Fibroblast cell subtypes in PPTC and APTC.

**S3. Additional Data**

S3.1 Expansion and Supplementary Content


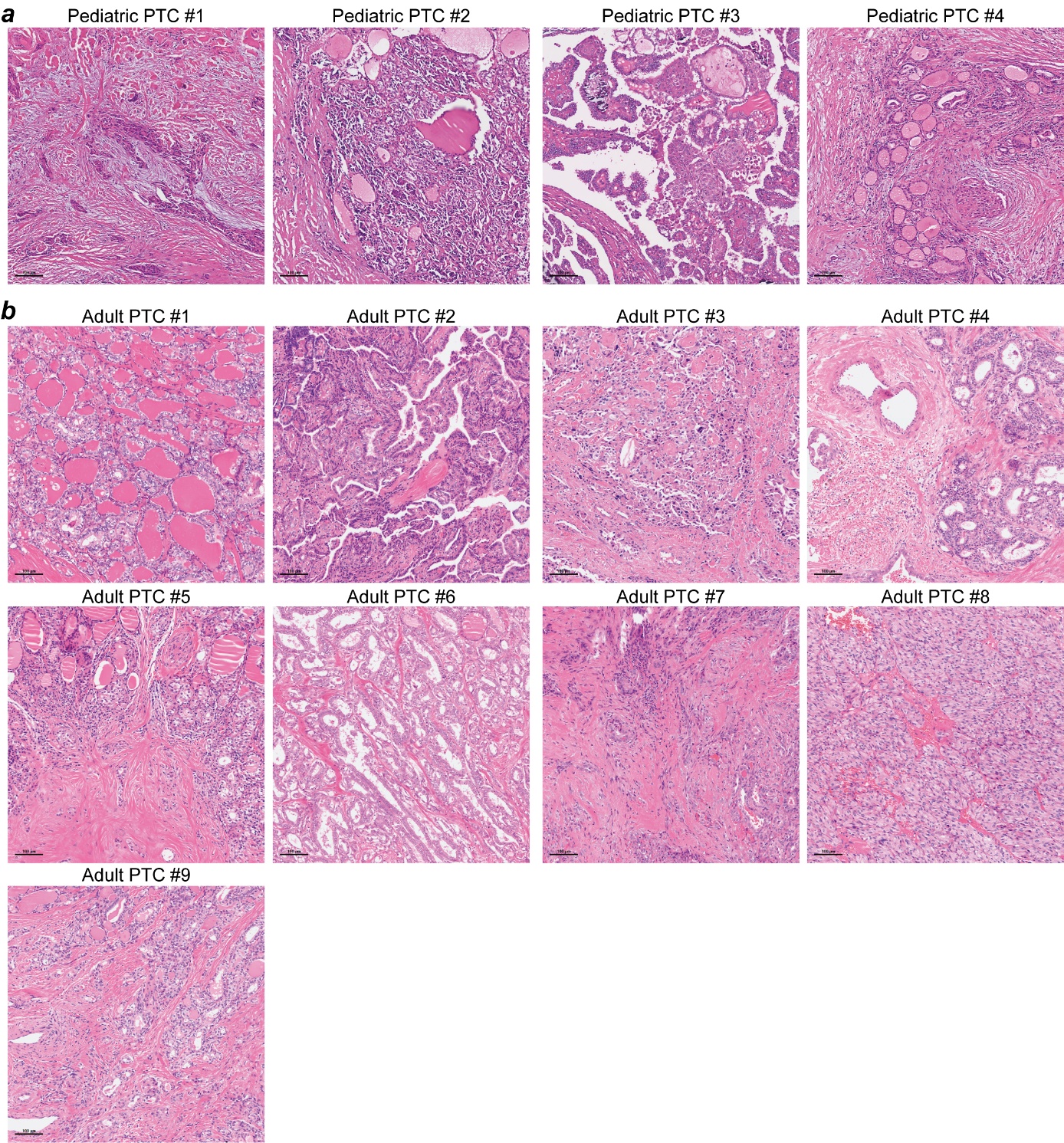


**Supplementary figure 1.** **Microscopic morphologies of PTC samples.** Representative H&E staining of tissue samples biopsied for scRNA-seq in pediatric **(a)** and **(b)** adult PTC tissues. Scale bar = 100μm.


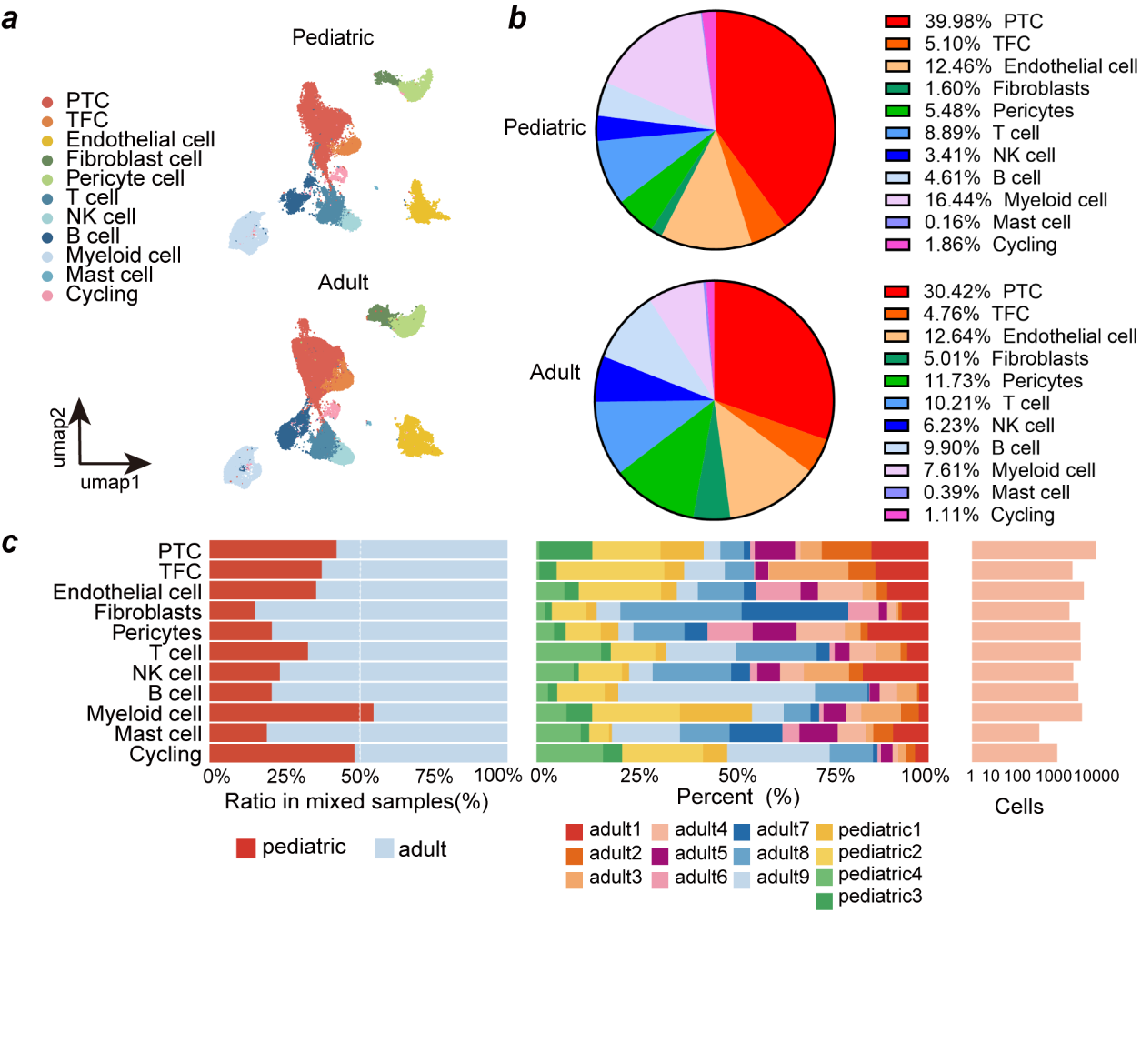


**Supplementary figure 2. Single-cell expression profiling of pediatric and adult PTC. (a)** UMAP plots illustrating the distribution of all cell types in pediatric and adult groups. **(b)** A pie chart shows the proportion of all cell types in pediatric and adult groups. **(c)** Bar plot indicating the fraction of annotated cell types originating from pediatric and adult PTC (left), as well as from each individual patient (middle); a histogram displaying the cell count of the annotated cell types (right).


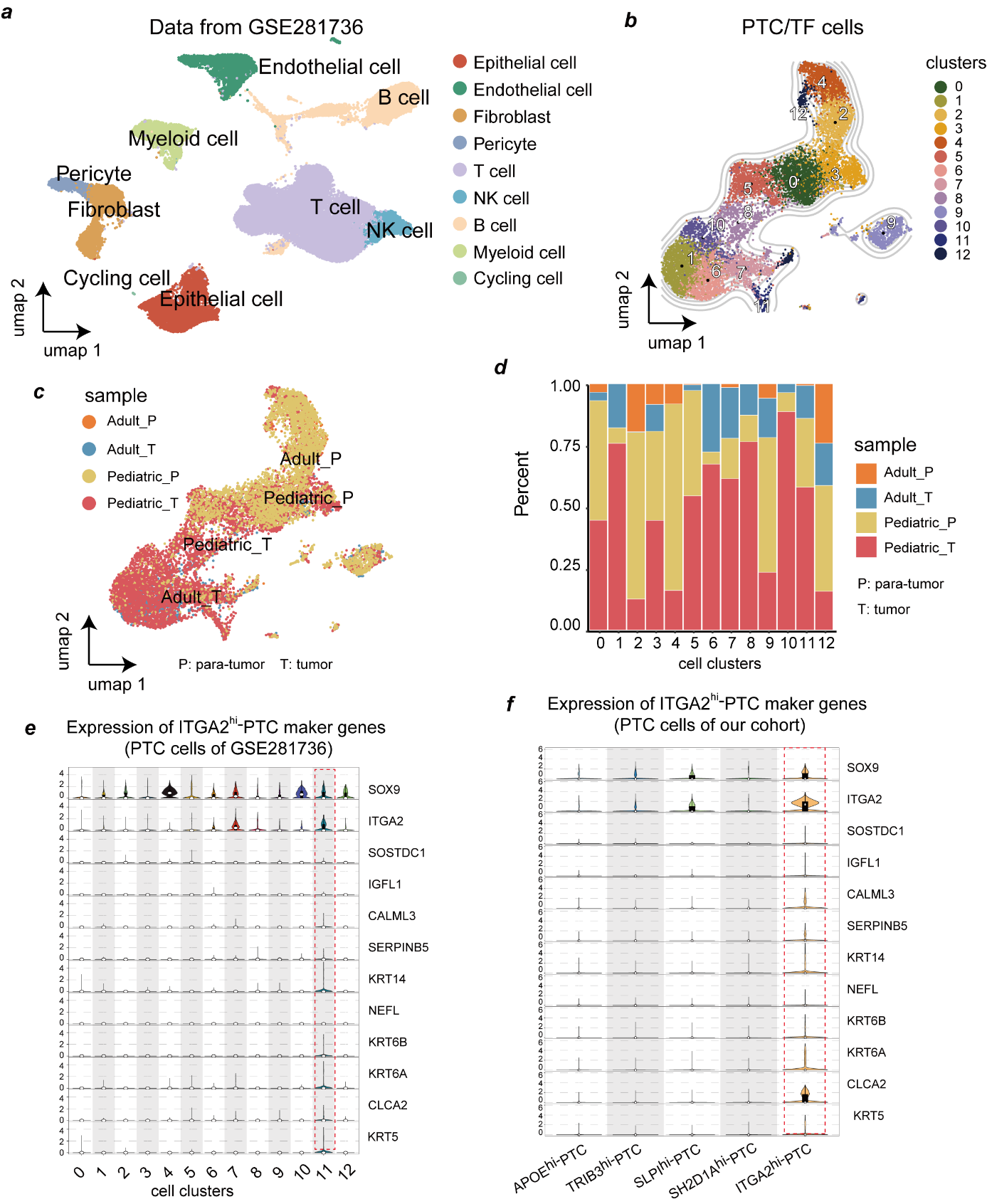


**Supplementary figure 3. Validation of the expression of ITGA2^hi^-PTC cell marker genes in GEO data. (a)** Downloaded the single-cell sequencing data for pediatric PTC included in GEO (GSE281736). Uniform Manifold Approximation and Projection (UMAP) visualization of 54,898 cells from 12 samples (cancer and adjacent tissues from one adult PTC and five pediatric PTC), depicting 9 cell types highlighted in different colors. **(b, c)** UMAPs showing the distribution of PTC/TF cell subtypes (**b**), and different sample groups (**c**). **(d)** Proportion of 13 PTC/TF cell subtypes shown in bar plots in different sample types. **(e)** Violin plots showing the expression distributions of ITGA2^hi^-PTC cell marker genes in 13 PTC/TF cell subtypes from GSE281736. **(f)** Violin plots showing the expression distributions of ITGA2^hi^-PTC cell marker genes in 5 PTC cell subtypes in our cohort. Adult_P, para-tumor tissue from adult patients; Adult_T, tumor tissue from adult patients, Pediatric_P, para-tumor tissue from pediatric patients, Pediatric_T, tumor tissue from pediatric patients.


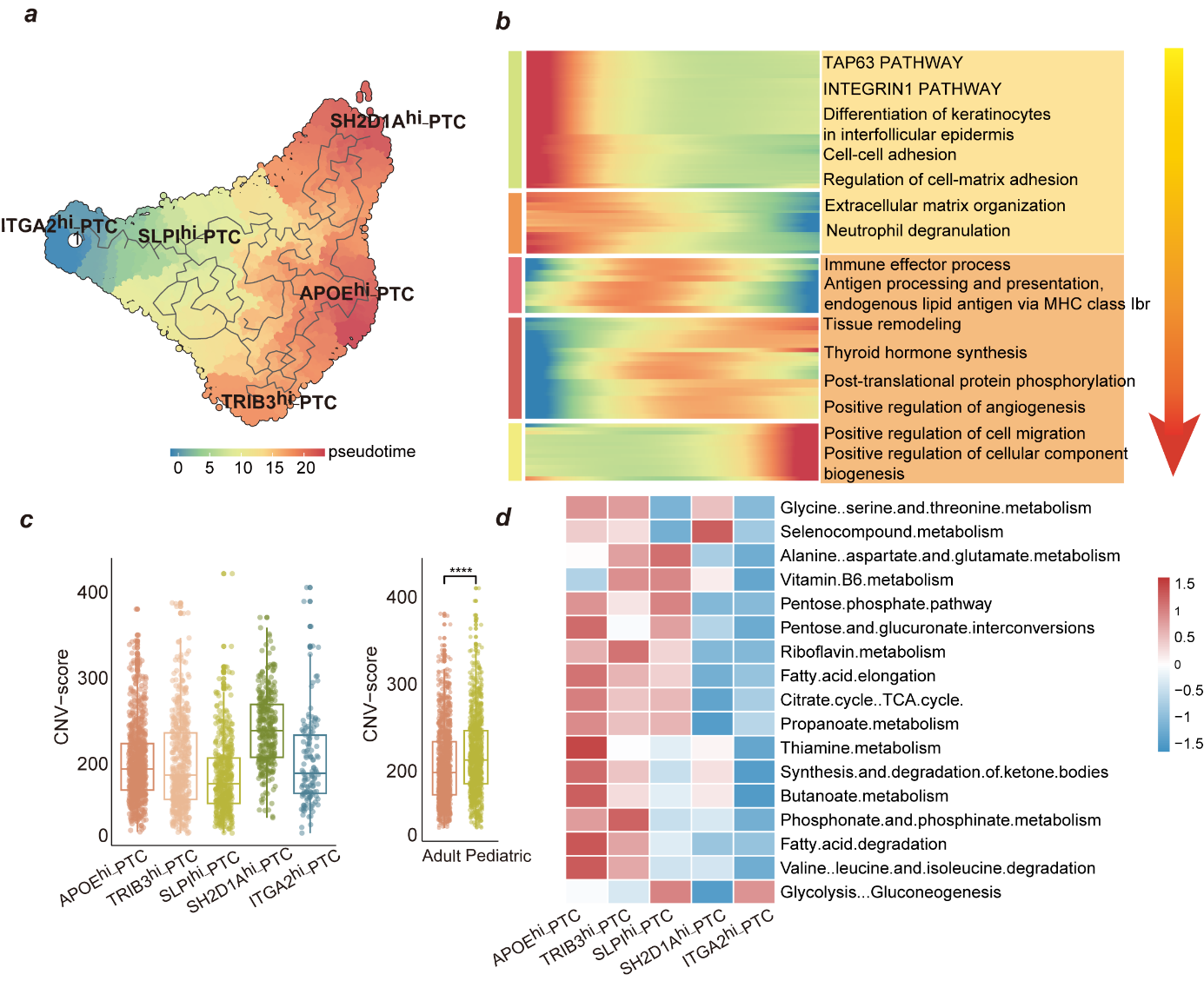


**Supplementary figure 4. Characteristics of heterogeneous cancer cell subtypes in thyroid cancer. (a)** UMAP projections of single-PTC cells along the inferred trajectory by Monocle3. Cells are colored by pseudotime. **(b)** Heatmap depicting the dynamics of differentially expressed genes (DEGs) and their enriched pathways along the trajectory. These DEGs were divided into five main clusters. **(c)** Box plots depicting the copy number variation (CNV) levels of annotated subclusters (left) and between pediatric (n=4) and adult groups (n=9) (right). The boxes represent the median (horizontal line) and the interquartile range (box), with Tukey-style whiskers extending beyond the box. *****p* < 0.0001, two-sided Student's t-test. **(d)** Heatmap illustrating the metabolic activity of all PTC subclusters analyzed using scMetabolism, (Scale bar ranging from −1.5 to 1.5).


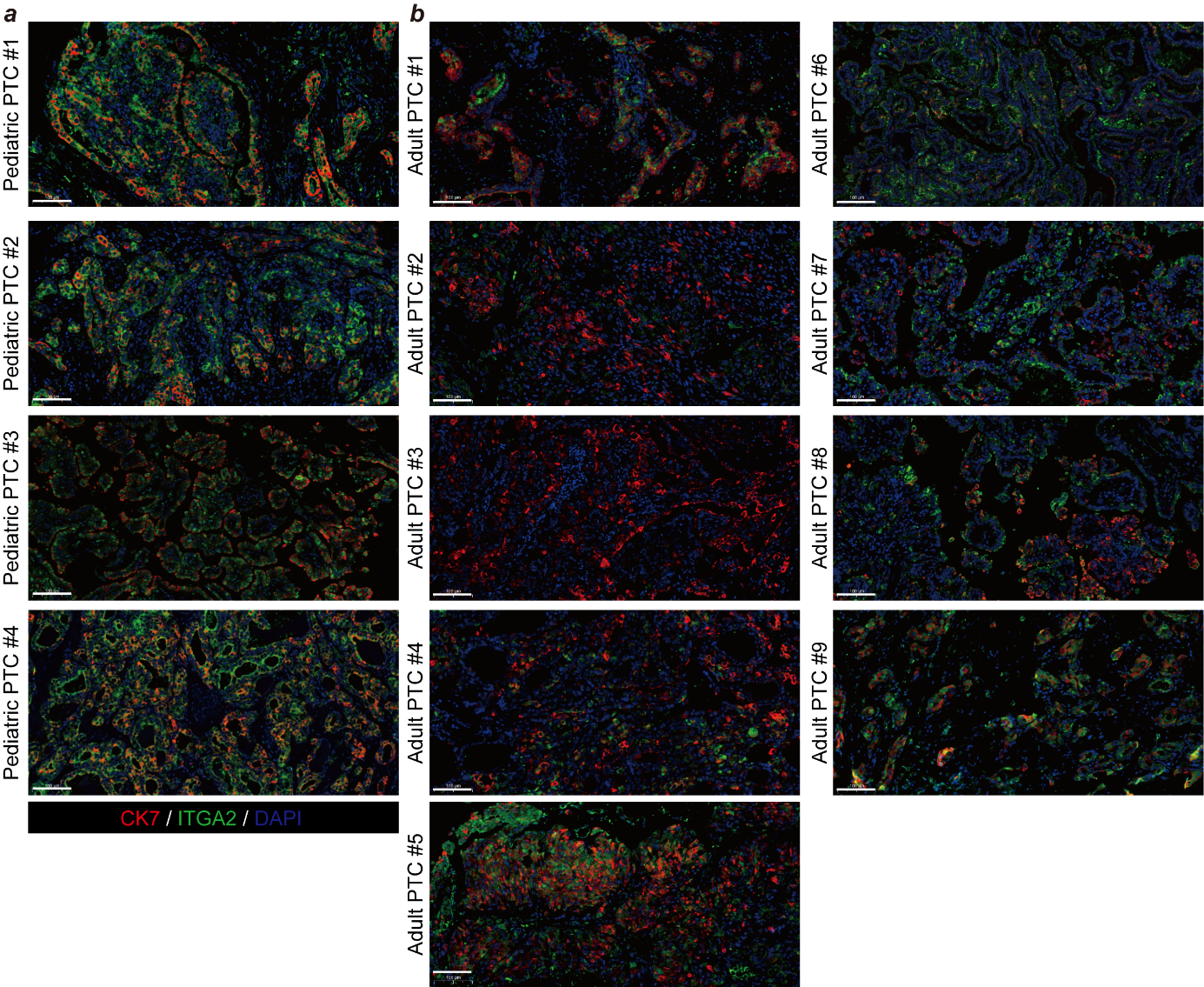


**Supplementary figure 5. mIHC staining of ITGA2^hi^-PTC cells in PTC samples.** Representative images of mIHC staining of ITGA2^hi^-PTC cells in pediatric **(a)** and **(b)** adult PTC samples. mIHC, multiplex immunohistochemistry. Scale bar=100 μm.

**
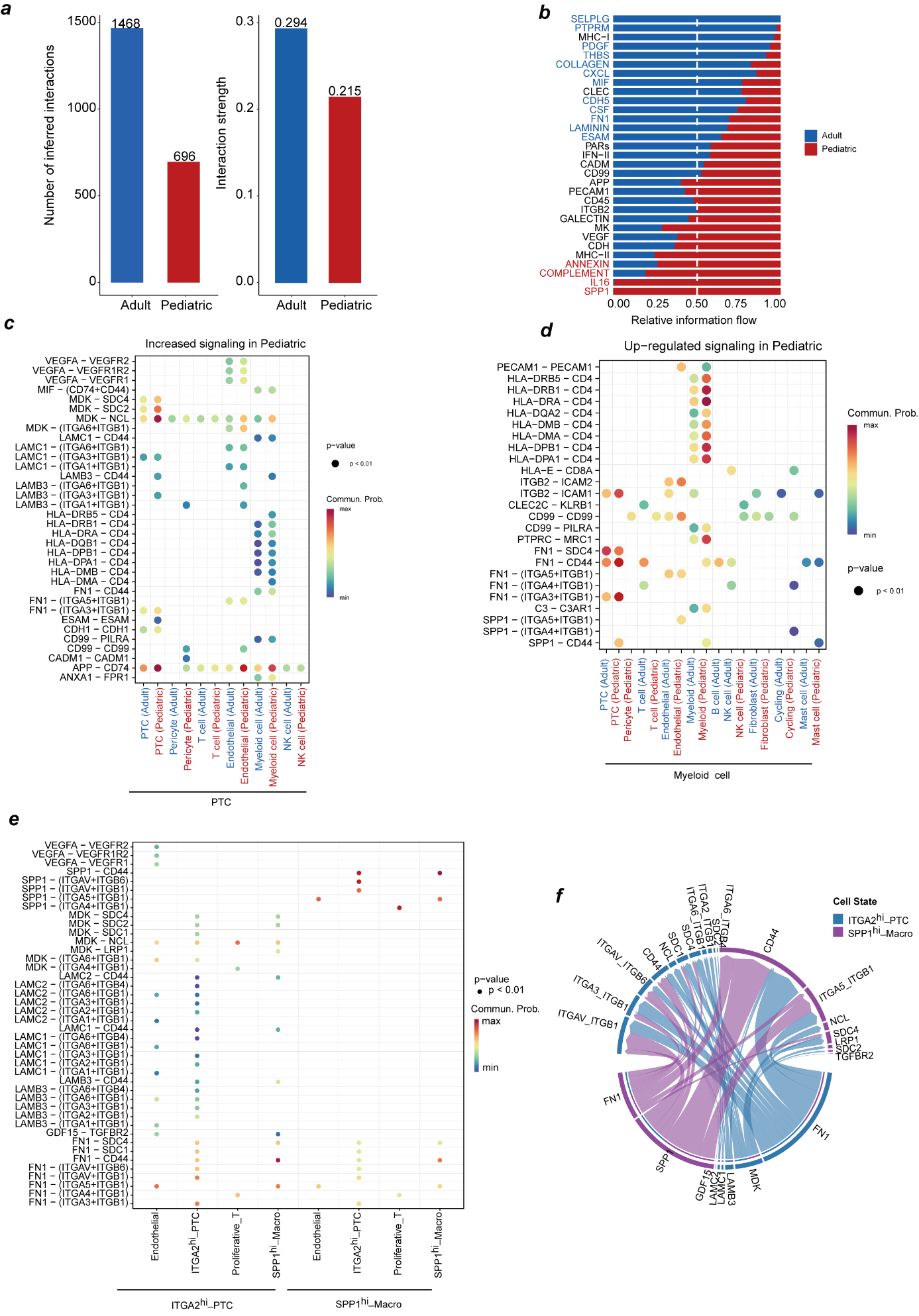
**

**Supplementary figure 6. Intercellular communication in PPTC and APTC revealed by CellChat. (a)** Bar plot illustrating the number and strength of intercellular interactions in both PPTC and APTC. **(b)** Bar plot depicting the relative activity of overall information flow between PPTC and APTC for the specified pathways. **(c-e)** Bubble plots highlighting the ​**​**differential interaction strength​​ of ligand-receptor pairs between specific cell types, including signals from PTC (c), Myeloid cells (d), ITGA2^hi^-PTC and SPP1^hi^-Macro(e). Dot color reflects communication probabilities, and dot size indicates computed p-values. Each cell group is color-coded. Empty space means the communication probability is zero. p-values are computed from a two-sided permutation test. **(f)** Circos plots show ligand and receptor pairs between ITGA2^hi^-PTC and SPP1^hi^-Macro.


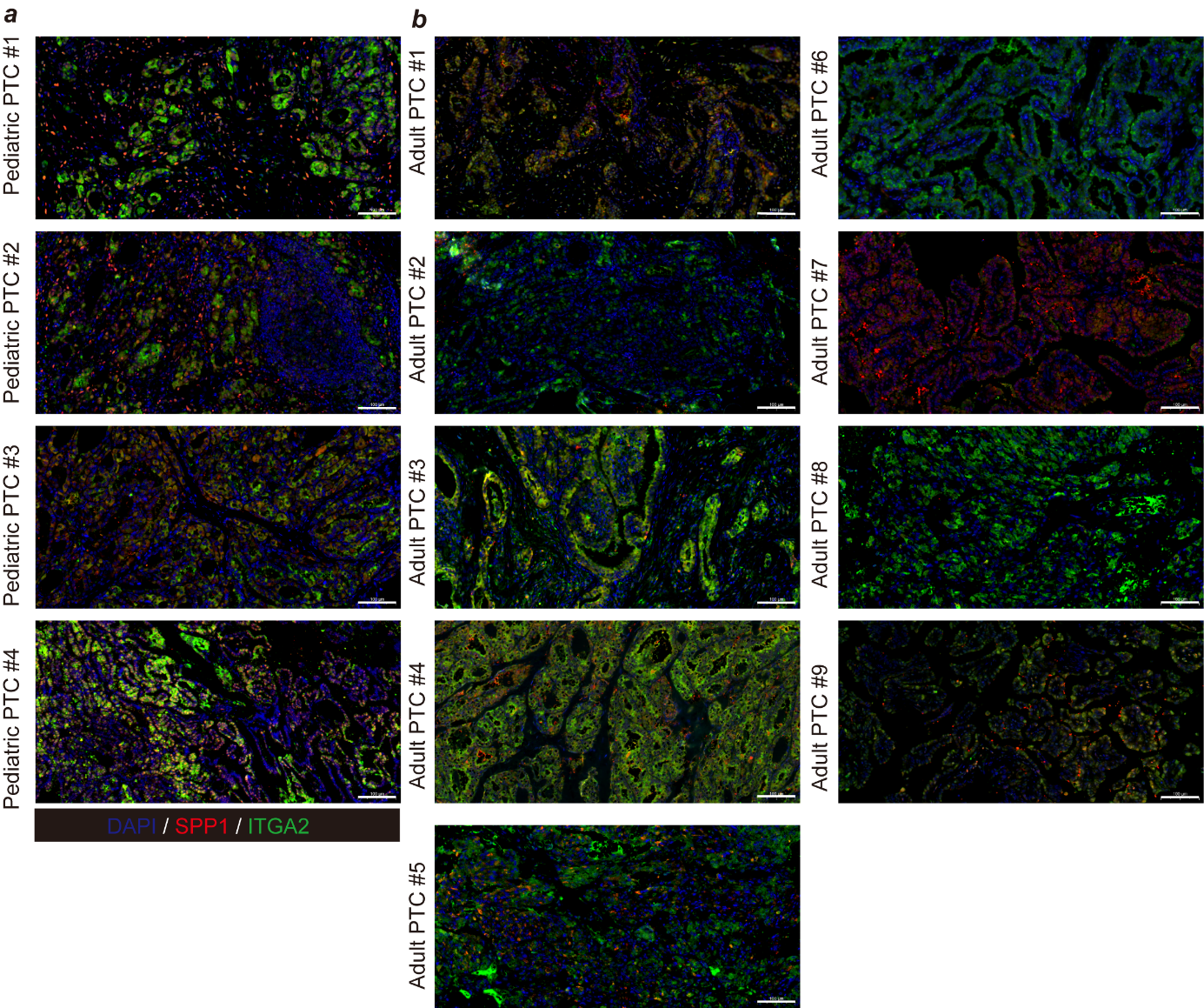


**Supplementary figure 7. mIHC staining reveals spatial co-localization of SPP1^hi^_macrophages and ITGA1^hi^_PTC cells in PTC samples.** Representative images of mIHC staining in pediatric **(a)** and **(b)** adult PTC samples. mIHC, multiplex immunohistochemistry. Scale bar=100 μm.

**S3.2 Supplementary Analysis**

**S3.2.1 Heterogeneity Landscape of T/NK Cells between PPTC and APTC**

As central effectors of anti-tumor immunity, T and NK cell functional states critically dictate immune efficacy. Through scRNA-seq analysis of 13,492 T/NK cells from PPTC and APTC, we identified 10 functionally discrete subsets, validated by spatial mapping (UMAP) and marker gene expression. These include CD4+ lineages (naïve CD4_Tn, exhausted CD4_Tex-CXCL13, and regulatory CD4_Treg-FOXP3/IL2RA), CD8+ compartments (naïve CD8_Tn, effector memory CD8_Tem-GZMK, exhausted CD8_Tex-CCL3), and rare populations including double-negative T cells (DNT_SATB1), proliferative T cells, and NK cells (**Fig. S8a, 8b, 8d**). Notably, PPTC displayed a distinct immunophenotype characterized by increased proliferative T cells and DNTs with reduced naïve and exhausted CD4+ T cells (**Fig. S8c**).

Functional multidimensional profiling across quiescence, regulation, proliferation, cytotoxicity, and exhaustion demonstrated that CD8_Tem-GZMK, CD8_Tex-CCL3, and NK cells exhibited cytotoxicity-exhaustion coupling, whereas proliferative T cells served as primary proliferation hubs (**Fig. S8e**). Strikingly, PPTC T/NK compartments showed attenuated exhaustion with enhanced proliferative capacity compared to APTC, suggesting sustained tumor-killing potential that may underlie superior pediatric prognosis. This was supported by elevated granzyme B (GZMB) expression in NK cells and increased Ki-67 levels in proliferative T cells (**Fig. S8f-8g**).

Mechanistically, proliferative T cells in PPTC upregulated T-cell differentiation regulators (DNTT, LEF1) and were enriched in lymphocyte differentiation pathways. Comparative functional scoring further confirmed their enhanced proliferation and cytotoxicity with reduced exhaustion relative to APTC counterparts (**Fig. S8h-8j**). Thus, these findings indicate that PPTC-specific proliferative T cells sustain functional T-cell pools within the tumor microenvironment, thereby driving potent anti-tumor immunity.


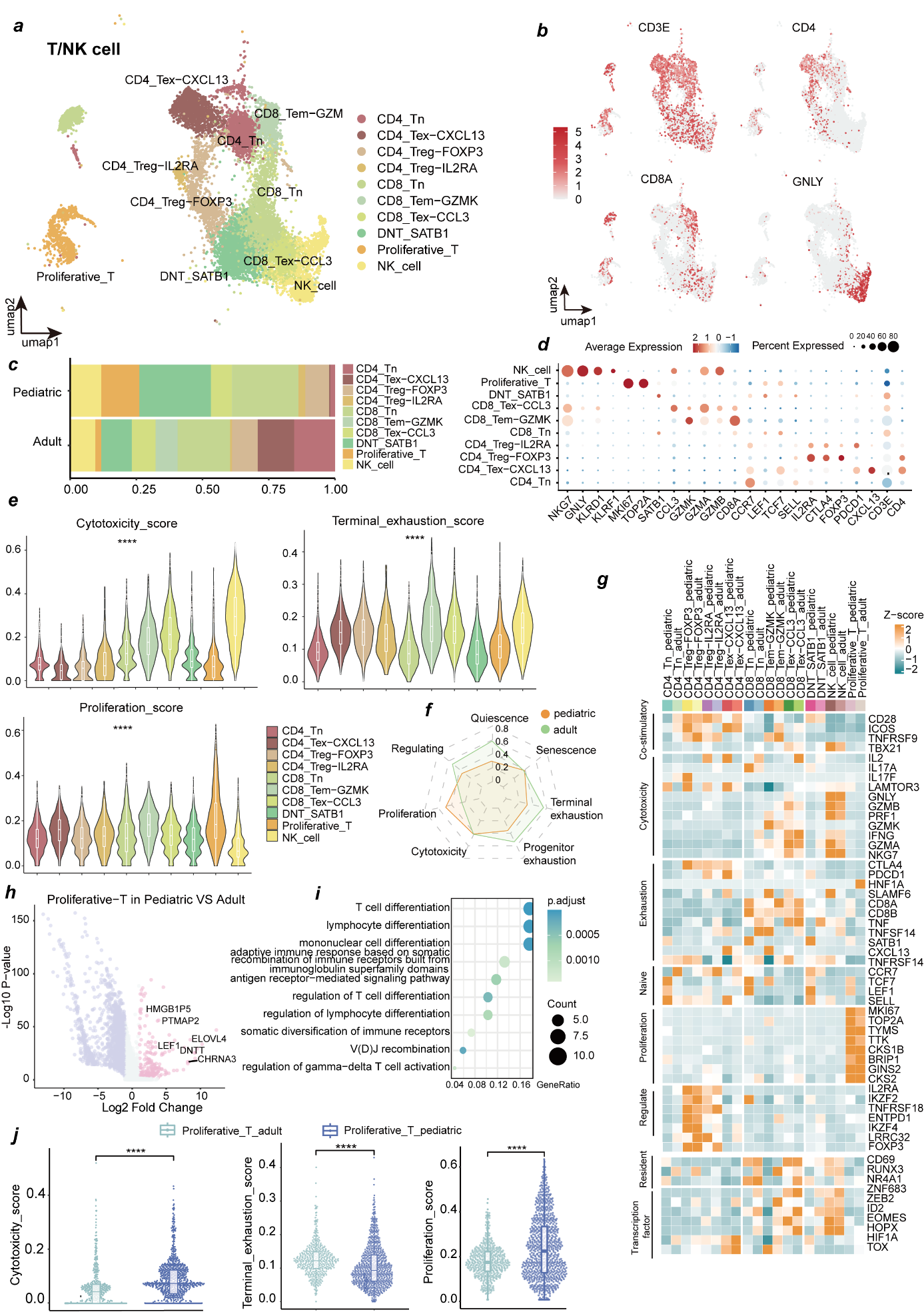


**Supplementary figure 8. T and NK cell subtypes in PPTC and APTC. (a)** UMAP of subclustered T/NK cells, labeled in different colors. **(b)** Feature plots showing the expression of selected specific genes. Cells with the highest expression level are colored red. **(c)** Bar plot showing the proportion of these annotated subclusters originating from PPTC and APTC. **(d)** Dot plots showing the expression of common marker genes across each T/NK cell subset. **(e)** Violin plots displaying the cytotoxicity, terminal-exhaustion, and proliferation scores in the selected subclusters. **** *p* < 0.0001, Kruskal–Walli’s rank-sum test was used. **(f)** Radar plots comparing the functional scores in PPTC and APTC. Gene set were obtained from TCellSI (T Cell State Identifier). **(g)** Heatmap showing the expression patterns of T/NK related functional genes in T/NK subsets of pediatric and adult patients. Color indicates the Z score scaled gene expression levels. **(h)** Volcano plot showing DEGs in proliferative-T in pediatric and adult samples. **(i)** Bubble chart showing the enrichment of specific pathways, based on upregulated genes in proliferative-T cells of PPTC. **(j)** Box plot showing the median (middle line), 25th, 75th percentiles (box) of cytotoxicity, terminal-exhaustion, and proliferation scores of proliferative-T in PPTC and APTC. Significance was determined using an unpaired two-sided Student’s t-test (*p* < 0.0001).

**S3.2.2 Heterogeneity Landscape of endothelial cells between PPTC and APTC**

Tumor angiogenesis sustains malignant progression by fueling metabolic demands of proliferation and metastasis. Through re-clustering of 11,348 endothelial cells, we delineated six functionally distinct subtypes: arterial (AE), venous (VE), capillary (Cap), and three tip cell phenotypes (Tip1-Tip3) (**Fig. S9a**). Notably, PPTC showed significant enrichment in VE (21.91% vs 19.05%), Cap (6.91% vs 4.03%), and Tip1 (31.56% vs 23.05%) subpopulations, whereas APTC was enriched in AE and Tip2 subtypes (**Fig. S9b**). Subtype-specific marker analysis identified AE via GJA4 and HEY1 expression, VE by ACKR1 and CLU upregulation, Cap/Tip1 by shared COL4A1 and PLVAP elevation, while Tip2 and Tip3 were defined by RND1 and IGFBP5 respectively (**Fig. S9c, 9d**). Importantly, PPTC-enriched Cap and Tip1 co-expressed pro-angiogenic receptors FLT1 and KDR, suggesting heightened angiogenic signaling in pediatric tumors (**Fig. S9e**).

PPTC endothelium displayed specialized immunomodulatory properties, characterized by upregulated chemokines CXCL4, CXCL4L2, and S100A2 versus APTC (**Fig. S9f**). Pathway analysis revealed enrichment of these genes in myeloid cell recruitment, lymphocyte trafficking, and NK chemotaxis pathways, while downregulated genes involved cytoplasmic translation processes (**Fig. S9g**). Thus, these findings indicate that PPTC endothelial cells actively orchestrate immune-microenvironment crosstalk through chemokine gradient establishment to recruit immunocytes.


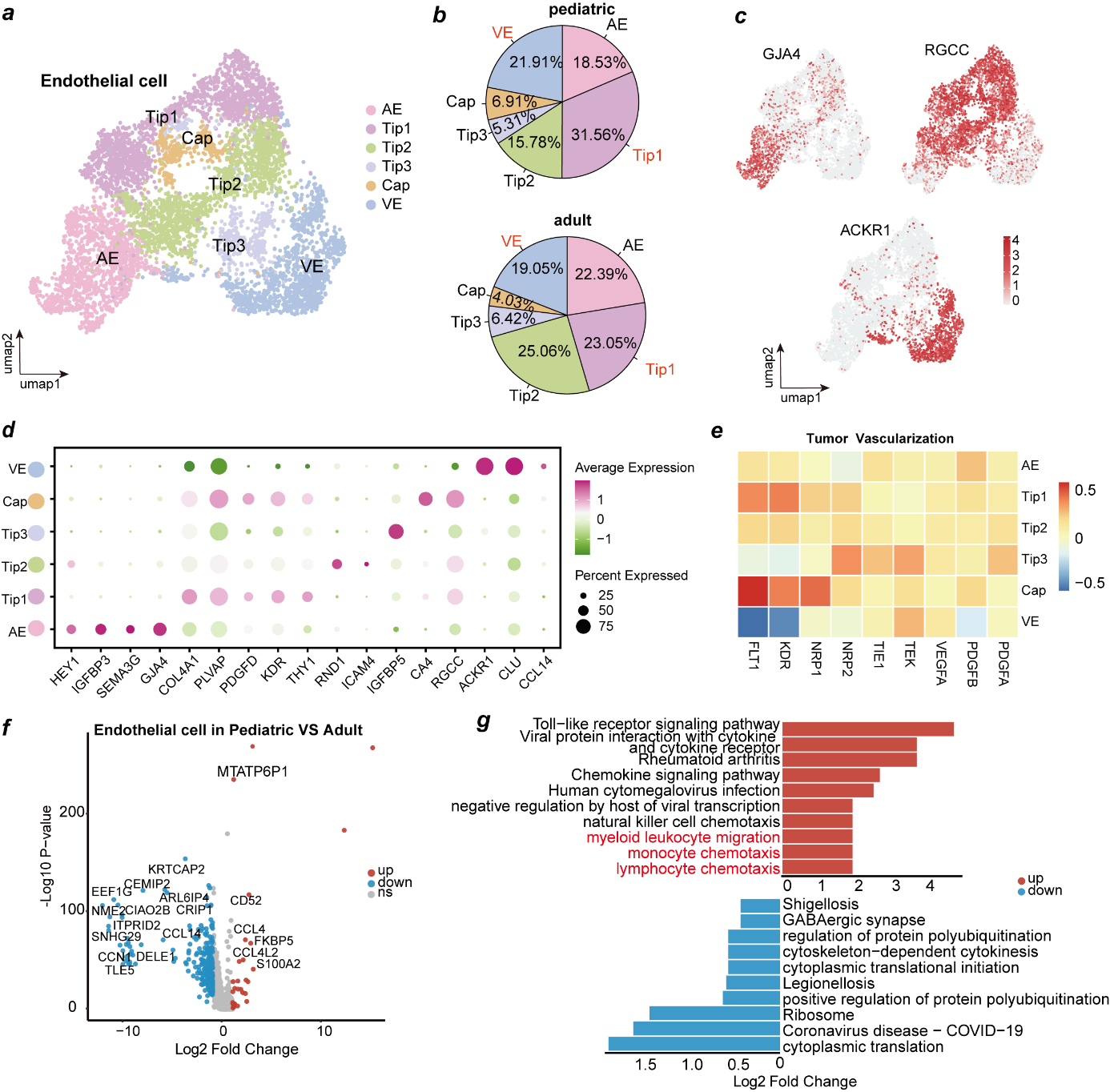


**Supplementary figure 9. Endothelial cell subtypes in PPTC and APTC. (a)** UMAP plot illustrating subclustered Endothelial cells, labeled in different colors. **(b)** Pie chart showing the percentage of the subclusters originating from pediatric and adult groups. **(c)** Feature plots showing the expression of selected specific genes. Cells with the highest expression levels are colored red. **(d)** Dot plots showing the expression of common marker genes in each subtype. **(e)** Heatmap showing the expression patterns of related tumor vascularization functional genes in Endothelial cell subsets. Color indicates the Z score scaled gene expression levels. **(f)** Volcano plot showing DEGs in Endothelial cells in pediatric and adult samples. **(g)** Bar chart showing the enrichment of specific pathways, based on the upregulated and downregulated genes, in PPTC Endothelial cells.

**S3.2.3 Heterogeneity Landscape of B cells between PPTC and APTC**

B cells orchestrate immunoregulatory functions within the tumor microenvironment through antibody production and antigen presentation. Comprehensive single-cell analysis of 7,211 B cells identified five functionally distinct subsets: naïve B (Naive_B), memory B (Memory_B), plasma B (Plasma_B), germinal center B (GC_B), and atypical memory B cells (Atypical_memory_B) (**Fig. S10a**). Striking compositional differences were observed between PPTC and APTC cohorts, with PPTC specimens exhibiting predominant populations of Naive_B, Plasma_B and Atypical_memory_B populations. In contrast, Memory_B cells were exclusively detected in Adult8/9 samples while GC_B cells were nearly restricted to Adult9 (**Fig. S10b, 10c**).

All subsets uniformly expressed canonical markers CD79A and MS4A1, yet displayed unique functional specialization: Plasma_B specifically upregulated the antibody-secreting gene MZB1, whereas Atypical_memory_B showed elevated expression of antigen-presentation regulator B2M (**Fig. S10d, 10e**). These distinct molecular signatures indicate subset-specific immunoregulatory roles that likely contribute to differential immune microenvironment landscapes between pediatric and adult tumors. The predominance of Plasma_B and Atypical_memory_B in PPTC further suggests enhanced humoral immunity and antigen-presentation capacity in pediatric cases.


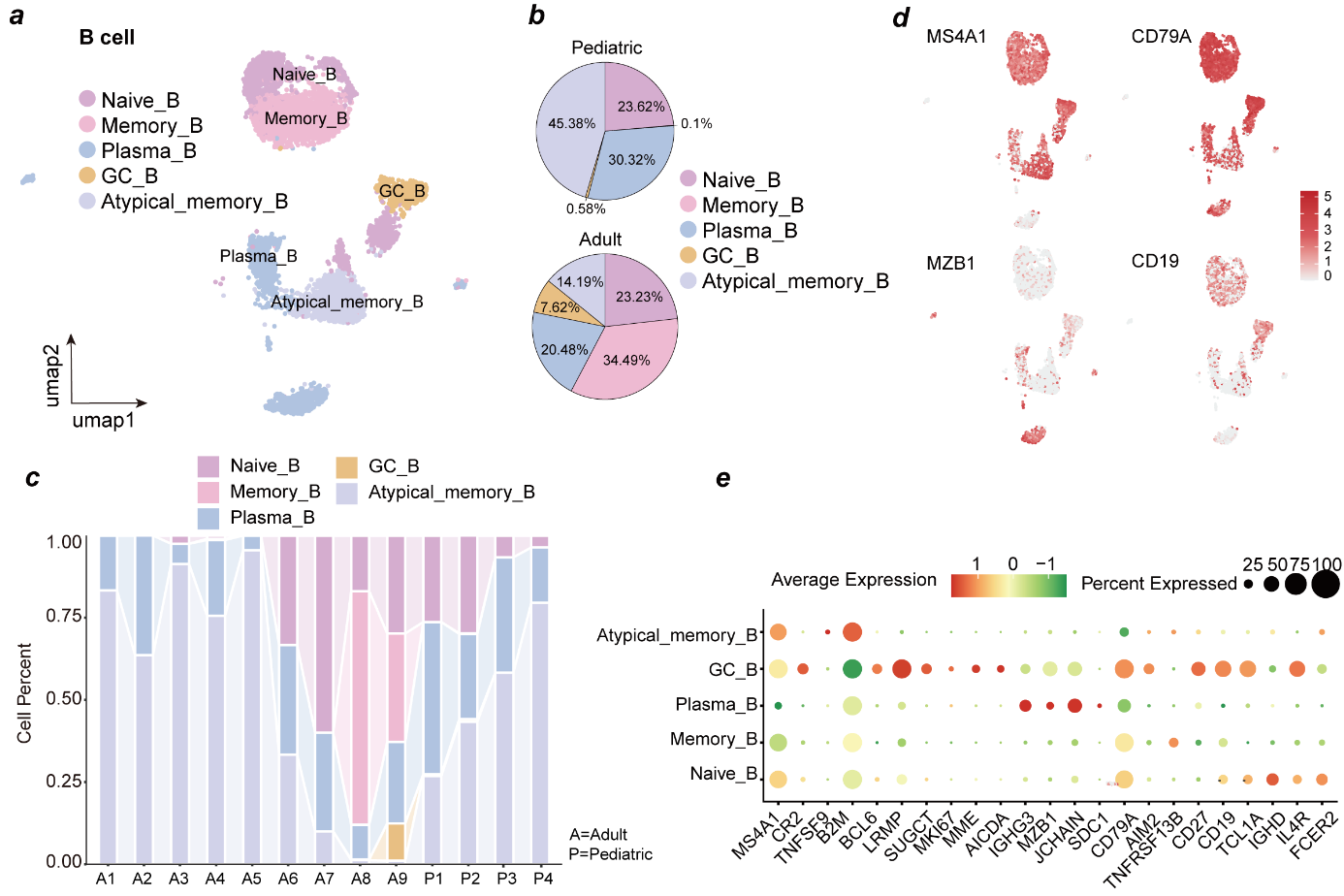


**Supplementary figure 10. B cell subtypes in PPTC and APTC. (a)** UMAP plot illustrating subclustered B cells, labeled in different colors. **(b)** Pie chart showing the proportion of the subtypes in pediatric and adult patients. **(c)** Bar plots showing the expression and proportion of the subtypes in each sample. **(d)** Feature plots showing the expression of selected specific genes. Cells with the highest expression levels are colored red. **(e)** Dot plots showing the expression of common marker genes in each B cell subtype.

**S3.2.4 Heterogeneity Landscape of stromal cells between PPTC and APTC**

Pericytes and fibroblasts constitute fundamental cellular components of the tumor extracellular matrix, critically influencing tumor progression, invasion, and metastasis. Single-cell clustering analysis of these stromal populations delineated eight functionally distinct subtypes: myoid pericytes (myoPC), conventional pericytes (PC), matrix pericytes (mPC), matrix-associated cancer-associated fibroblasts (mCAF), inflammatory CAFs (iCAF), smooth muscle cells (SMC), vascular-associated CAFs (vCAF), and proliferative CAFs (pCAF) (**Fig. S11a**). Molecular characterization revealed subtype-specific signatures: PCs expressed contractile protein RGS5, while CAF subtypes exhibited differential specialization—iCAFs secreted inflammatory cytokine IL6, mCAFs overexpressed extracellular matrix gene POSTN, vCAFs expressed angiogenesis-related FLT1, and pCAFs showed enrichment of proliferation marker MKI67 (**Fig. S11b, 11d**).

Comparative analysis demonstrated significant distribution differences between pediatric (PPTC) and adult (APTC) tumor microenvironments. PPTC specimens exhibited markedly higher proportions of proliferative pCAFs, whereas APTC showed dominance of iCAFs and mCAFs (**Fig. S11c, 11e**). Functional profiling indicated enhanced proliferative capacity in PPTC pCAFs, contrasting with stronger inflammatory signaling in APTC iCAFs and matrix-remodeling functions in APTC mCAFs (**Fig. S11f**). These findings demonstrate distinct stromal reprogramming patterns: PPTC microenvironments feature pCAF-driven proliferative dominance, while APTC relies on iCAF/mCAF-mediated inflammatory signaling and matrix remodeling pathways to support tumor progression.


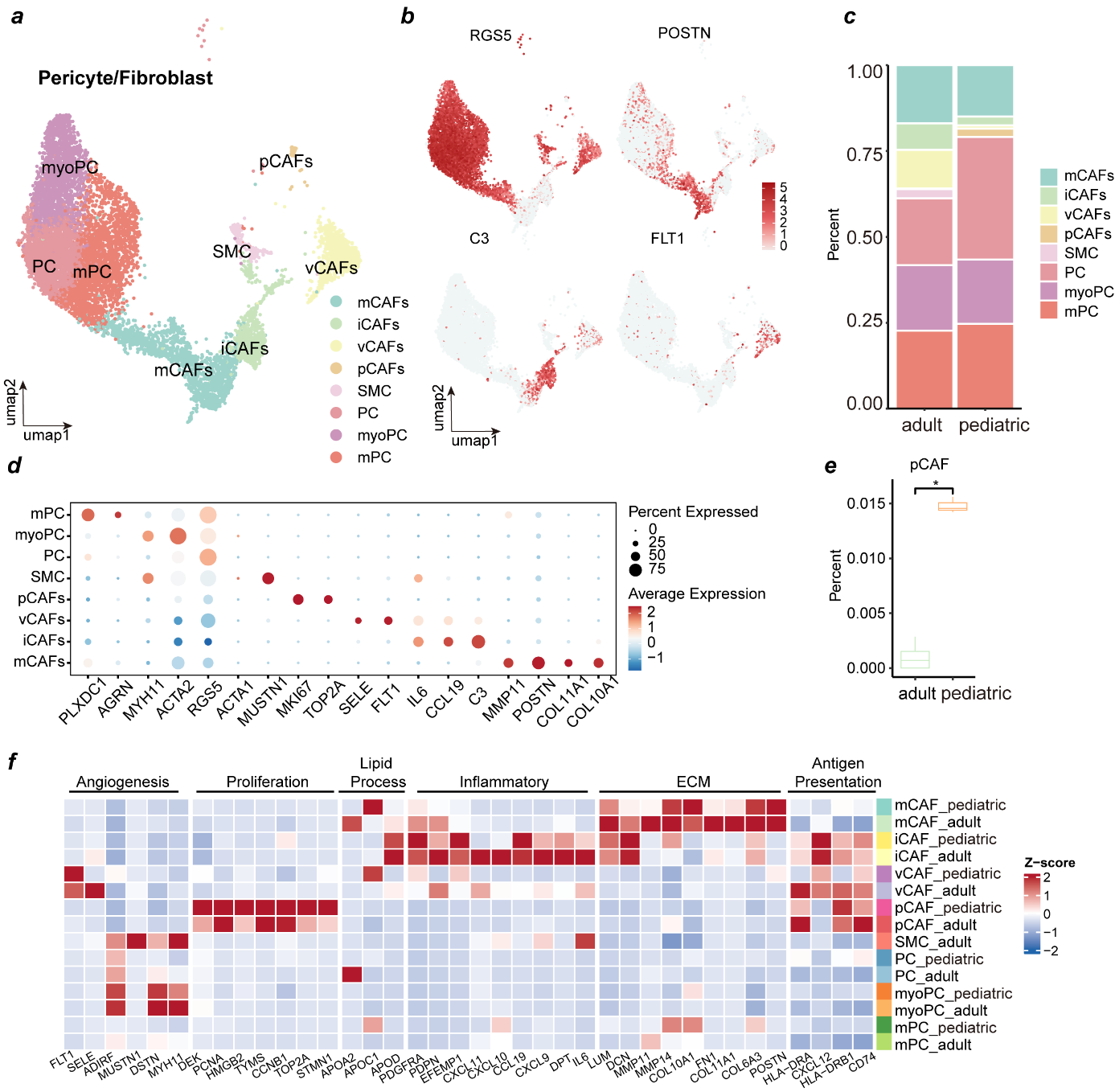


**Supplementary figure 11. Pericyte/Fibroblast cell subtypes in PPTC and APTC. (a)** UMAP plot illustrating subclustered Pericyte/Fibroblast cells, labeled in different colors. **(b)** Feature plots showing the expression of selected specific genes. Cells with the highest expression levels are colored red. **(c)** Bar plots showing the proportion of the subtypes in PPTC and APTC. **(d)** Dot plots showing the expression of common marker genes in each Pericyte/Fibroblast cell subtype. **(e)** Box plots showing the proportion of pCAF in PPTC (n=4) and APTC (n=9). Significance was determined using Wilcoxon rank-sum test (*p* < 0.05). **(f)** Heatmap showing the expression patterns of Pericyte/Fibroblast-related functional genes in Pericyte/Fibroblast subsets of pediatric and adult patients. Color indicates the Z score scaled gene expression levels.
